# Ergolide Regulates Microglial Activation and Inflammatory-Mediated Dysfunction: A Role for the Cysteinyl Leukotriene Pathway

**DOI:** 10.3390/ijms26115050

**Published:** 2025-05-23

**Authors:** Danielle M. Galvin, Sara Fernandez-Garcia, Emma Dawson, Ciara Pryce, Billy P. Egan, Niamh C. Clarke, Alison L. Reynolds, Derek A. Costello

**Affiliations:** 1 UCD School of Biomolecular & Biomedical Science, University College Dublin, D04 V1W8 Dublin, Ireland; danielle.galvin@ucdconnect.ie (D.M.G.); billy.egan@ucdconnect.ie (B.P.E.); niamh.clarke4@ucdconnect.ie (N.C.C.); 2 UCD Conway Institute, University College Dublin, D04 V1W8 Dublin, Ireland; alison.reynolds@ucd.ie; 3 UCD School of Veterinary Medicine, University College Dublin, D04 W6F6 Dublin, Ireland

**Keywords:** TLR, NFκB, microglia, nitric oxide, cytokines, zebrafish larvae

## Abstract

Neurodegenerative diseases are characterised by the progressive loss of neurons, leading to a decline in specific brain functions. Alzheimer’s disease (AD) and Parkinson’s disease (PD) are the most prevalent, affecting approximately 60 million people worldwide. The pathogenesis of these diseases is complex, combining inflammatory, oxidative, and excitotoxic processes that result in neuronal dysfunction and death. Despite recent advances, there is currently no cure for AD and PD. Available therapies demonstrate limited efficacy, highlighting the continuing need for novel therapeutic approaches. Ergolide, a naturally occurring sesquiterpene lactone from the *Inula brittanica* plant, has shown immunoregulatory properties in systemic immune cells and potential applications in certain cancers. This study examines whether the therapeutic effects of ergolide extend to the brain. We explored its mechanisms of action in vitro, and its capacity to restore behavioural integrity in zebrafish models of inflammation and neurotoxicity in vivo. We report the ability of ergolide to attenuate inflammatory cytokine and nitric oxide (NO) production from microglia in response to toll-like receptor activation. We further propose a role for the NFκB and cysteinyl leukotriene (CysLT) pathways in ergolide-mediated regulation of microglial activation. Ergolide did not protect against oxidative-induced neuronal death in vitro or mitigate seizure activity in zebrafish. Instead, we revealed a pro-oxidant and cytotoxic effect on neuroblastoma cells. Importantly, ergolide improved survival and alleviated the dysfunction in sensorimotor behaviour in a zebrafish model of inflammation. Our findings reveal a neuroprotective effect of ergolide, likely stemming from its immunoregulatory capacity. We also support further investigation of the CysLT pathway as a therapeutic target for neuroinflammatory-related disease.

## 1. Introduction

Neurodegenerative diseases, such as Alzheimer’s disease (AD) and Parkinson’s disease (PD), are typified by the progressive loss of neuronal integrity in specific brain regions, leading to gradual decline in function. Despite differences in their onset, aetiology, and symptomatology, many age-related neurodegenerative diseases are characterised by similar pathological features. These include chronic neuroinflammation associated with dysfunctional activation of microglia, as well as oxidative stress due to imbalance in the generation of reactive oxygen and nitrogen species, which underlie subsequent excitotoxic and ferroptotic cell death. Together, these complex processes result in the neuronal dysfunction and gradual brain atrophy that characterise the disease pathogenesis [1,2]. Despite decades of research, the search for a cure or disease-modifying therapies remained elusive. Although recent developments in immunotherapeutic approaches have begun to show great promise, particularly among the AD community, current treatments remain restricted in their use and efficacy [3,4]. This highlights the necessity to identify novel strategies that can target the individual pathological processes that contribute to disease progression.

Toll-like receptors (TLRs) are a family of pattern recognition receptors. In the CNS, they are most abundantly expressed in glial cells, and to a lesser extent in neurons. Under healthy conditions, they play an important homeostatic role in the brain, regulating processes including synaptic pruning, neurogenesis, and synaptic plasticity. This largely occurs through mediation of neurotrophic factor production and by stimulating the phagocytic capacity of microglia [5]. Chronically dysfunctional microglia are a key feature of neurodegenerative disease, characterised by their sustained activation and uncontrolled inflammatory response. This leads to persistent release of proinflammatory cytokines, such as interleukin (IL)-1β, TNFα, and IL-6, along with neurotoxic factors, including reactive oxygen species (ROS) and nitric oxide (NO); this is consistent with the classical ‘M1’ proinflammatory activation state. Together, these factors promote the mitochondrial and DNA damage that ultimately results in neuronal death [1]. Microglial-expressed TLRs are widely regarded as primary drivers of inflammation associated with neurodegeneration. In particular, the roles of TLR2 and TLR4 have been highly reported, largely due to their ability to recognise the endogenous neurotoxic peptides, such as β-amyloid (Aβ) and α-synuclein (αS), that accumulate in the AD and PD brain, respectively [6]. Indeed, our group, among multiple others, contributed to our understanding of the integral role of TLR activation in mediating the inflammatory changes and neuronal dysfunction associated with disease states [7,8,9,10,11,12,13,14]. TLRs elicit their response via regulation of several transcription factors, including AP1, NFκB, and interferon regulatory factor (IRF). In particular, NFκB is considered the master transcription factor driving inflammation, promoting the production of pro-inflammatory mediators such as cytokines and chemokines, and contributing to the generation of ROS and NO. In addition, NFκB regulates the transcription of inflammatory-associated molecules, including the NOD-like receptor, NLRP3. Assembly of the NLRP3 inflammasome further promotes activation of caspase-1, IL-1β, and IL-18, which are key contributors to neurodegenerative pathology and further support the role of the TLR/ NFκB/NLRP3 axis in neurodegenerative disease [15,16]. Upregulation of NFκB activation is reported in the neurodegenerating brain, and accordingly, its inhibition has proven beneficial to resolving functional deficits in experimental models of AD and PD [17,18,19,20]. This inflammatory signal is predominantly mediated via the mitogen-activated protein (MAP) kinases JNK and p38 as the primary route of NFκB activation downstream of TLRs [21,22]. Consequently, modulation of MAP kinase activity has also shown promise for targeting the consequences of neurodegenerative disease [23,24,25].

Inflammatory mediators such as pro-inflammatory cytokines and oxygen radicals are generated by CNS cells in response to injury, infection, or disease. Increased levels of TNFα, IL-6, IL-1β, and MCP1 have been reported in the CSF of AD and PD patients [26,27,28,29] and susceptibility to neurodegenerative disease [30]. Inflammatory cytokines and chemokines are among the earliest indicators of neuropathology, but only account for a portion of the chemical mediators that regulate the inflammatory response. The role of lipids and lipid derivatives, in particular eicosanoid imbalance, gained attention in regulation of neuroinflammation, neuronal dysfunction, and cognitive impairment [31]. Recent findings from our own laboratory, among others, also reveal the potent impact of saturated and unsaturated fatty acids on the priming and regulation of microglial and macrophage activation [7]. Primarily, products of arachidonic acid metabolism, such as prostaglandins and leukotrienes, have been implicated in the pathogenesis of several neurodegenerative states. For example, prostaglandin PGE2 has been highlighted as a contributor to the neuronal loss associated with both AD [32] and PD [33], while the leukotriene, LTA4, is reported to enhance the toxicity induced by Aβ [34].

Cysteinyl leukotrienes (CysLTs) are lipid mediators, derived from the further metabolism of LTA4, that play a well-known role in respiratory inflammation, including asthma and allergic rhinitis [35,36]. There are three members of the CysLT family, LTC4, LTD4, and LTE4, that evoke cellular responses through interaction with the G-protein-coupled receptors CysLTR1 and CysLTR2. While the CysLT pathway is well understood in the context of respiratory inflammatory conditions, its potential involvement in cerebral conditions has begun to receive interest in recent years. LTD4 has the highest affinity for both receptors [36], and is reported to activate microglial cells, leading to increased phagocytosis, as well as TNFα and IL-1β release [37,38]. Moreover, the selective CysLTR2 agonist NMLTC4 has also been shown to bias microglia towards a pro-inflammatory phenotype [39]. Elevated CysLT expression has been observed in the CSF of multiple sclerosis (MS) patients [40], and their catalytic enzyme 5LOX increased in lesions from a mouse model of the disease [41]. Accordingly, the antagonism of CysLTR1 has proven to be beneficial for alleviating leukocyte infiltration to the CNS and consequent demyelination in the experimental autoimmune encephalomyelitis (EAE) mouse model of MS [42]. This extends further to rescuing the cognitive impairment in streptozotocin-induced models of neuroinflammation [43,44], along with anti-convulsant and neuroprotective effects in response to pentylenetetrazol (PTZ)-induced seizure [45]. LTD4 was reported to promote Aβ generation and neurotoxicity in a mouse model of AD via CysLTR1-mediated activation of the NFκB pathway. Antagonising CysLTR1 in turn mitigated the resultant cognitive deficit [46,47]. More recently, overexpression of CysLTR1 in the hippocampus was found to accelerate cognitive and synaptic dysfunction and amyloidosis in APP/PS1 AD transgenic mice [48]. Collectively, these studies implicate the CysLT pathway, largely via CysLTR1 in the neurodegenerative processes characteristic of AD. Targeting the CysLT pathway therefore presents a promising strategy for therapeutic intervention into AD pathology.

For centuries, naturally occurring agents and their derivatives have been exploited for their diverse therapeutic properties. Modern scientific validation further highlighted their therapeutic potential, particularly in the context of neurodegenerative diseases [49]. Notable among these are agents that demonstrate anti-inflammatory and antioxidant properties [49,50]. For example, we, among others, previously reported on the neurotherapeutic potential of flavones and flavonoid derivatives via their capacity to regulate microglial activation [8,51]. Terpenes are a large group of naturally occurring hydrocarbons comprised of repeating units of isoprene. Terpenes, including turmerones, β-caryophyllene, and limonene, all revealed anti-inflammatory properties, yielding neuroprotective effects in various in vitro and in vivo models of disease [52,53,54,55,56]. Ergolide is a sesquiterpene lactone isolated from the *Inula brittanica* plant from the Asteraceae family, known for anti-inflammatory, anti-tumour, antimicrobial, and antioxidant activities [57]. In particular, ergolide has been found to mitigate NFκB activation in RAW 264.7 cells, a murine macrophage/monocyte cell line, which in turn ameliorated the production of PGE2 [58]. Ergolide has also been reported to induce apoptosis of Jurkat T cells via a similar mechanism [59]. Further analysis revealed that ergolide likely attenuates NFκB activation through both prevention of its nuclear translocation via stabilisation of IκB, and direct interference with its DNA-binding capacity [58]. Recently, these effects have been shown to extend to models of osteoarthritis, in which ergolide alleviated the inflammatory response in chondrocytes in vitro and in vivo via mitigation of the NFκB pathway [60]. Ergolide has also been identified as an anti-proliferative agent, evoking cytotoxicity of tumour cells through ROS-mediated apoptosis [61]. In further support of its anti-cancer properties, ergolide has proven effective at reducing the survival of metastatic uveal melanoma cells, both in culture and in a zebrafish xenograft model. Interestingly, this appears to be largely independent of its immunomodulatory actions and further interrogation is required to fully elucidate this mechanism [62].

Throughout recent years, zebrafish (*Danio rerio*) have become an increasingly attractive tool with which to model features of human neurological and neurodegenerative disease [63,64,65]. This largely stems from the generation of functional nervous and immune systems during their larval stages of development, rendering them effective for recapitulating the neuroinflammatory and neurotoxic characteristics of disease [64,66,67]. Such models have proven particularly useful for the discovery of novel neurotherapeutics [67,68]. To that end, the current study sought to determine whether the therapeutic potential of ergolide may extend to the brain. We set out to evaluate the anti-inflammatory and antioxidant capacity of ergolide in microglia and neurons in vitro. We further explored its cellular mechanism of action, particularly examining the role for the CysLT pathway. Moreover, we examined whether ergolide can restore behavioural integrity in zebrafish models of inflammation and neuronal dysfunction in vivo.

## 2. Results

**Figure 1:**
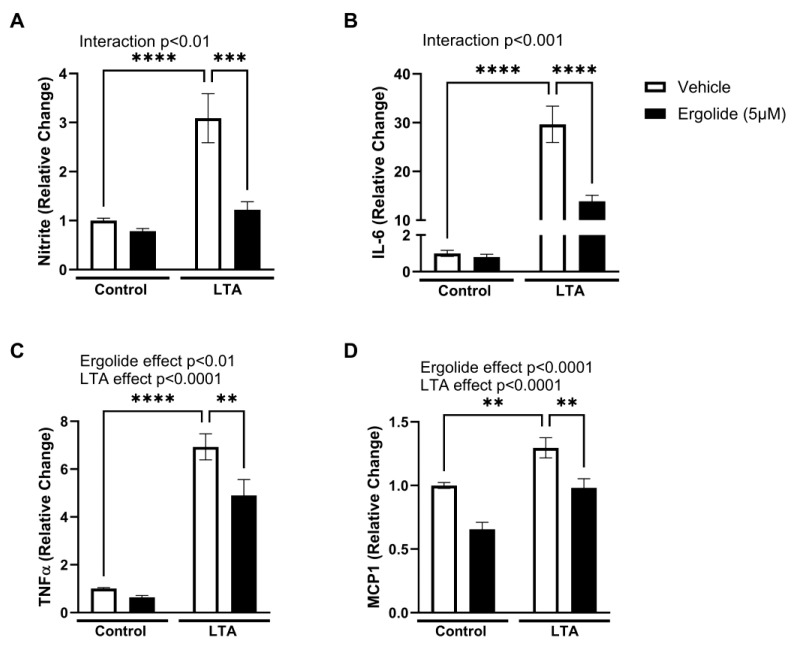
Ergolide attenuates LTA-induced expression of inflammatory mediators. Nitrite (Griess assay) (**A**), IL-6 (**B**), TNFα (**C**), and MCP1 (ELISA) (**D**) were measured in a supernatant of BV2 cells treated with LTA (5 µg/mL) in the presence of ergolide (5 µM) or DMSO (0.025%; 24 h). Data are expressed as a proportion of control and presented as mean ± SEM (*n* = 9–21 replicates from 3–7 independent experiments). Statistical comparisons were made using two-way ANOVA followed by Tukey’s post hoc analysis (** *p* < 0.01, *** *p* < 0.001, and **** *p* < 0.0001).

Murine BV2 cells are a well-characterised in vitro model of microglia. Their response to TLR activation shares close homology to that of primary cultures [69], and reliably replicates the polarisation of microglial activation in vitro [8,70]. The responsiveness of BV2 cells to the TLR2 agonist lipoteichoic acid (LTA) has been well documented [7,8,71]. Recognising the important role of TLR2 in mediating the inflammation associated with neurodegenerative conditions, we used LTA-stimulated BV2 cells as an in vitro model to investigate how the sesquiterpene lactone ergolide regulates the inflammatory changes relevant to the brain. To determine microglial activation towards an ‘M1’-like state, we measured the release of pro-inflammatory mediators from LTA-stimulated BV2 cells. Ergolide has previously been reported to regulate NFκB activation and the production of inflammatory mediators in vitro, at concentrations ranging from 1 to 10 µM [58,59]. Having established a maximum tolerable concentration of 5 µM in BV2 cells, cells were incubated with LTA (5 µg/mL) for 24 h in the presence and absence of ergolide (5 µM) or DMSO (0.025%) as a vehicle control. As previously reported, here, we confirm a significant increase in nitrite (*p* < 0.0001; Figure 1A), indicative of NO production, cytokines IL-6 (*p* < 0.0001; Figure 1B) and TNFα (*p* < 0.0001; Figure 1C), and the chemokine MCP1 (*p* < 0.01; Figure 1D) in LTA-stimulated cells. The significant enhancement in nitrite upon LTA exposure was reduced in by ergolide (*p* < 0.001; Figure 1A). A similar reduction in LTA-induced TNFα, IL-6, and MCP1 expression was also seen in the presence of ergolide at 5 µM (Table 1, Figure 1).

**Figure 2:**
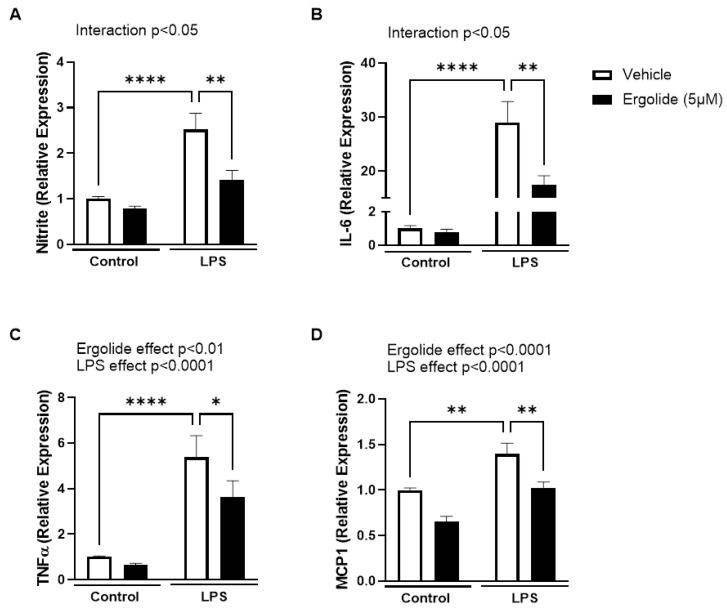
LPS-induced activation of BV2 cells is alleviated by ergolide. Nitrite (**A**), IL-6 (**B**), TNFα (**C**), and MCP1 (**D**) were measured in the supernatant collected from BV2 cells treated with LPS (5 ng/mL) for 24 h in the presence of ergolide (5 µM) or a vehicle control (DMSO, 0.025%). Data are expressed as a proportion of control and presented as mean ± SEM (*n* = 9–21 replicates from 3–7 independent experiments). * *p* < 0.05, ** *p* < 0.01, **** *p* < 0.0001; and two-way ANOVA followed by Tukey’s post-tests.

Ergolide has previously been reported to reduce inflammatory signalling in LPS/IFNγ-stimulated (M1) macrophages [58]. To determine whether the effects in microglia are restricted to TLR2-mediated responses, we examined its effect on BV2 cells stimulated with the TLR4/MD2 agonist LPS (5 ng/mL; 24 h). In a similar manner to LTA, exposure to LPS significantly increased expression of nitrite (*p* < 0.0001; Figure 2A), IL-6 (*p* < 0.0001; Figure 2B), TNFα (*p* < 0.0001; Figure 2C), and MCP1 (*p* < 0.01; Figure 2D) compared with controls. In all cases, the LPS-induced changes were significantly attenuated in the presence of ergolide (Table 1, Figure 2).

**Figure 3:**
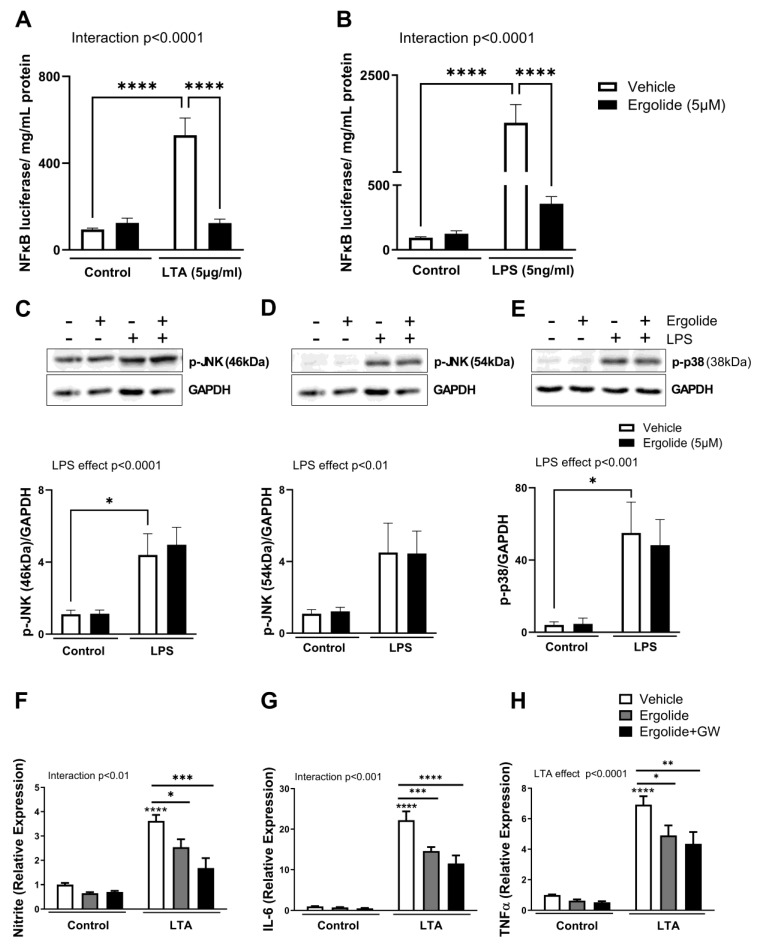
Ergolide-mediated NFκB inhibition is independent of MAP kinase and PPARγ. THP1-Lucia™ cells were treated with ergolide (5 µM) or a vehicle, along with either LTA (5 µg/mL; (**A**)) or LPS (5 ng/mL; (**B**)), for 24 h. Luciferase expression was measured and expressed relative to total protein content determined by BCA assay (*n* = 25 replicates from 7 independent experiments). BV2 cells were pre-treated with ergolide for 30 min and stimulated with LPS (200 ng/mL) for a further 30 min. Expression of p-JNK 46 kDa (**C**) and 54 kDa (**D**) and p-p38 (**E**) was assessed by Western immunoblot as a proportion of GAPDH expression. *n* = 9 replicates from 3 independent experiments. BV2 cells were incubated with LTA (5 μg/mL) in the presence and absence of ergolide (5 µM) ± GW9662 (10 µM) for 24 h. Supernatant expression of nitrite (**F**) IL-6 (**G**) and TNFα (**H**) was quantified. *n* = 8–12 replicates from three to seven independent experiments. Inserts illustrate representative immunoreactive bands corresponding to p-JNK, p-p38, and GAPDH. Data are presented as mean ± SEM and analysed using a two-way ANOVA followed by Tukey’s post hoc test (* *p* < 0.05, ** *p* < 0.01, *** *p* < 0.001, and **** *p* < 0.0001).

Inflammatory changes induced by activation of TLR2 and TLR4 are largely mediated by the transcription factor NFκB. To better understand the cellular mechanisms through which ergolide can alleviate cytokine and NO production, we sought to investigate its impact on the activation of NFκB in response to both LTA and LPS. We therefore carried out an NFκB activity assay using THP1 Lucia™ cells that express a luciferase reporter controlled by NFκB [72]. As expected, NFκB activation was significantly upregulated by stimulation with both LTA (5 µg/mL; *p* < 0.0001; Figure 3A) and LPS (5 ng/mL; *p* < 0.0001; Figure 3B). In both cases, this was significantly reduced in the presence of ergolide (*p* < 0.0001; Figure 3). To further assess the signal leading to NFκB activation, we examined the expression of phosphorylated JNK and p38 MAP kinase relative to GAPDH in LPS-stimulated BV2 microglia. LPS exposure for 30 min enhanced the expression of both p-JNK and p-p38 compared with controls (*p* < 0.05; Figure 3C,E). Interestingly, preincubation with ergolide did not alter this change (Figure 3C,E), suggesting that ergolide likely targets NFκB via a MAP kinase-independent mechanism.

Activation of PPARγ has been widely reported to mediate the neuroprotective effects of naturally occurring agents [73,74], along with attenuation of TLR-NFκB-dependent inflammatory changes [75,76,77]. To determine whether ergolide may exert its effects through a similar pathway, the specific PPARγ antagonist, GW9662 (10 µM), was applied to LTA-stimulated BV2 cells in combination with ergolide. However, ergolide continued to reduce the LTA-induced expression of nitrite (*p* < 0.001; Figure 3F), IL-6 (*p* < 0.0001; Figure 3G), and TNFα (*p* < 0.01; Figure 3H) despite the presence of GW9662. This suggests that the anti-inflammatory effects of ergolide are independent of PPARγ activation.

**Figure 4:**
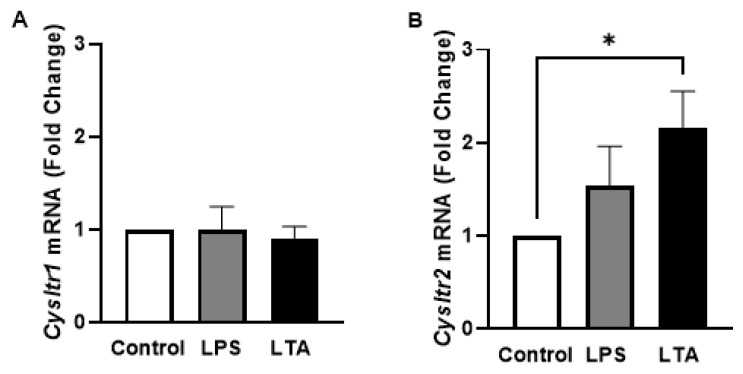
LTA enhances mRNA expression of *Cysltr2* in BV2 cells. BV2 cells were exposed to LPS (5 ng/mL) or LTA (5 μg/mL) for 24 h. Expression of *Cysltr1* (**A**) and *Cysltr2* (**B**) mRNA was evaluated relative to *Gapdh* using qPCR. Data are presented as mean ± SEM (*n* = 5-8 independent experiments). * *p* < 0.05, one-way ANOVA followed by Dunnett’s post hoc test.

CysLTs are lipid mediators of inflammation. Although predominantly known for their role in allergy and respiratory disorders, they have more recently been explored for their role in neuroinflammatory-associated diseases [78,79]. In an effort to further elucidate the mechanism of action of ergolide in microglia, we sought to examine the potential role of the CysLT pathway. BV2 cells were treated with the LPS or LTA for 24 h and the expression *Cysltr1* and *Cysltr2* mRNA was measured. Neither LPS nor LTA had any effect on the expression of *Cysltr1* (Figure 4A). However, expression of *Cysltr2* was increased in stimulated cells, reaching significance in cells exposed to LTA compared with untreated controls (*p* < 0.05; Figure 4B).

**Figure 5:**
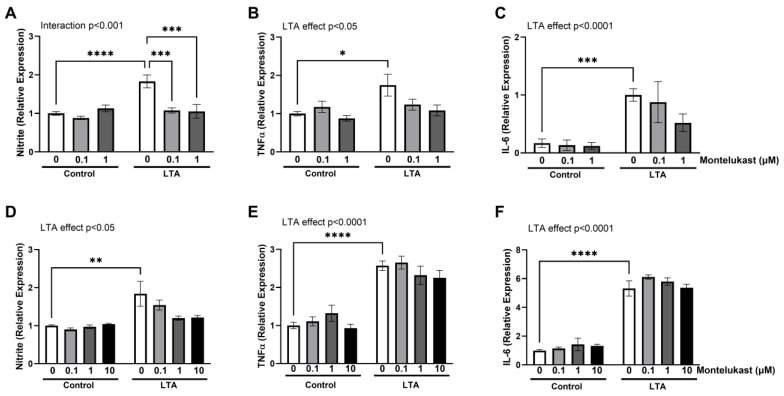
CysLTR1 antagonism does not robustly reduce LTA-induced activation of BV2 or N9 cells. BV2 cells (**A**–**C**) and N9 cells (**D**–**F**) were treated with LTA (5 µg/mL) for 24 h in the presence and absence of montelukast (0.1, 1.0, or 10 µM). Expression of nitrite (**A**,**D**), TNFα (**B**,**E**), and IL-6 (**C**,**F**) was measured in cell supernatant following 24 h incubation. Data are expressed as the proportion of control or LTA and presented as mean ± SEM (*n* = 6–18 replicates from 3-6 independent experiments). Statistical analysis was conducted using a two-way ANOVA followed by Tukey’s post hoc test (* *p* < 0.05, ** *p* < 0.01, *** *p* < 0.001, and **** *p* < 0.0001).

**Figure 6:**
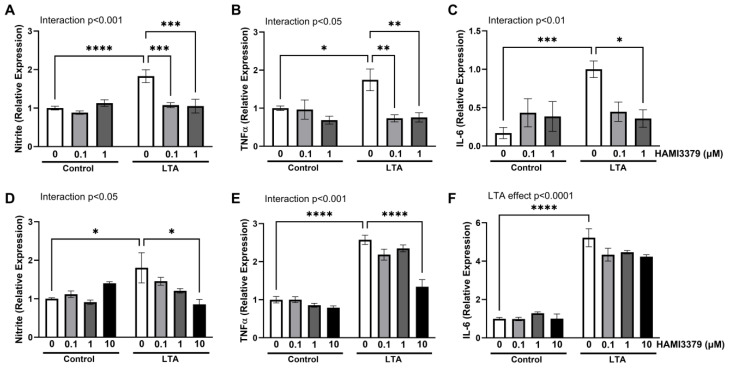
The CysLTR2 antagonist HAMI3379 reduces LTA-induced microglial activation. BV2 cells (**A**–**C**) and N9 cells (**D**–**F**) were treated with LTA (5 µg/mL) for 24 h in the presence or absence of HAMI3379 (0.1, 1.0, or 10 µM). Supernatant expression of nitrite (**A**,**D**), TNFα (**B**,**E**), and IL-6 (**C**,**F**) was determined. Data are presented as a proportion of control or LTA. All data are expressed as mean ± SEM (*n* = 6–18 replicates from 3-6 independent experiments). Statistical comparisons were made with two-way ANOVA followed by Tukey’s post hoc analysis (* *p* < 0.05, ** *p* < 0.01, *** *p* < 0.001, and **** *p* < 0.0001).

To examine whether activation of CysLT receptors plays a role in TLR-mediated production of inflammatory mediators, BV2 cells were stimulated with LTA in the presence and absence of the CysLTR1 antagonist, montelukast (0.1 and 1 µM). The LTA-induced release of nitrite was significantly attenuated by montelukast at 0.1 µM and 1 µM (*p* < 0.0001; Figure 5A); however, this was not reflected as a consistent reduction in the expression of either TNFα (Figure 5B) or IL-6 (Figure 5C). To confirm that this finding is not specific to BV2 cells, the experiment was repeated in N9 microglia. Exposure to LTA resulted in an increase in nitrite, TNFα, and IL-6, in a similar manner to that observed in BV2 cells (Figure 5D–F, respectively). However, co-application of montelukast at 0.1, 1, or 10 µM did not alter this effect. We therefore sought to assess the role of CysLTR2, and repeated the experiment using the specific CysLTR2 antagonist HAMI3379 (HAMI). HAMI significantly alleviated the LTA-induced increase in all inflammatory markers (Figure 6A–C), suggesting that CysLTR2 activation mediates the effects of LTA. A similar attenuation in the production of nitrite and TNFα was observed in LTA-stimulated N9 cells exposed to 10 µM HAMI (Figure 6D,E). Although a modest effect of HAMI was also seen on LTA-induced IL-6 expression, this was not significant (Figure 6F). Taken together, our findings highlight a prominent role for CysLTR2 in regulating the TLR-mediated inflammatory response in microglia.

**Figure 7:**
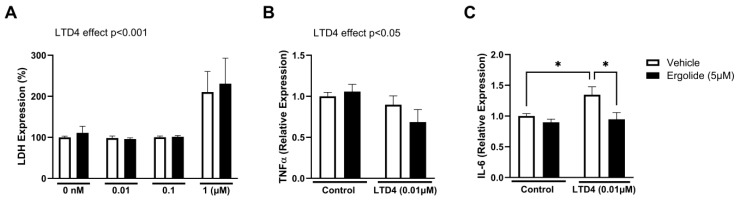
Ergolide reduces LTD_4_-induced IL-6 expression in BV2 microglia. BV2 cells were exposed to LTD4 (0.01, 0.1, or 1 µM), in the presence of either ergolide (5 μM) or a vehicle for 24 h. Cytotoxicity was assessed by supernatant expression of LDH (**A**) and presented as a percentage of control (mean ± SEM). Supernatant concentrations of TNFα (**B**) and IL-6 (**C**) were measured by ELISA. Data are expressed as a proportion of the control and presented as mean ± SEM (*n* = 8–12 replicates from 4-6 independent experiments). Statistical comparisons were made by two-way ANOVA followed by Tukey’s post hoc tests (* *p* < 0.05).

Having established that blocking CysLTR2 can alleviate microglial activation in a similar manner to ergolide, we next investigated whether the effects of ergolide may arise due to modulation of this pathway. Leukotriene D_4_ (LTD4) is a CysLT that is known to mediate inflammatory effects in vascular and respiratory systems through activation of both CysLTR1 and CysLTR2 [80]. Therefore, we evaluated the response of BV2 cells to LTD4 in the presence and absence of ergolide. LTD4 promoted cytotoxicity of BV2 cells at 1 µM (*p* < 0.001; 24 h), which was not seen at either 0.01 or 0.1 µM (Figure 7A). However, ergolide did not ameliorate this cytotoxic effect (Figure 7A). In light of its known role in mediating inflammation, we assessed microglial cytokine production in response to LTD4 at 0.01 µM, which was well-tolerated by the cells. Interestingly, we observed no increase in TNFα; in fact, a modest but significant reduction in expression was measured (*p* < 0.05; Figure 7B). However, IL-6 was significantly increased in LTD4-treated BV2 cells compared with controls (*p* < 0.05). Moreover, this was significantly restored in the presence of ergolide (*p* < 0.05; Figure 7C). These findings further support a role for CysLTR activation in the regulation of microglial function, albeit a more restricted response than that induced by TLRs. However, we also highlight the ability of ergolide to inhibit this, suggesting that the anti-inflammatory effects of ergolide may be, at least in part, due to regulation of the CysLT pathway.

**Figure 8:**
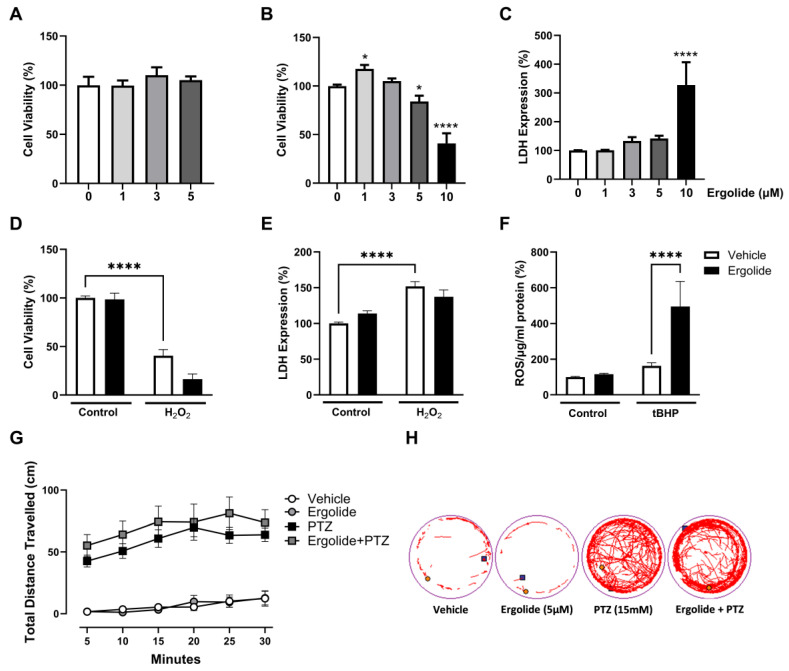
Ergolide promotes neuronal toxicity in vitro and does not alleviate seizure activity in vivo. N2a and SH-SY5Y cells were treated with either ergolide (N2a: 1, 3, 5 µM; SH-SY5Y: 1, 3, 5, and 10 µM) or DMSO control for 24 h. Cell viability was evaluated in N2a (**A**) and SH-SY5Y cells (**B**), and cytotoxicity of SH-SY5Y cells was determined by LDH expression (**C**). *n* = 4–28 replicates from 2–7 independent experiments. SH-SY5Y cells were incubated with H_2_O_2_ (0.5 mM; 24 h) in the presence and absence of ergolide (3 µM). Cell viability (**D**) and LDH (**E**) were assessed. N2a cells were pre-incubated with ergolide (5 µM; 30 min) prior to application of tBHP (200 µM; 4 h). ROS generation was determined as a proportion of total protein concentration, determined by BCA assay (**F**). *n* = 6–21 replicates from 3-7 independent experiments. Data are expressed as mean ± SEM. (**G**) Zebrafish larvae (4 dpf) were exposed to ergolide (5 μM) or DMSO (0.025%), followed by application of PTZ (15 mM) to the bathing media. Locomotor activity was monitored for 30 min using the Zebrabox VideoTrack System, and total distance travelled was measured. Data are expressed as mean distance travelled per 5 min ± SEM, *n* = 8 larvae per group. (**H**) Representative swimming trajectory of 1 larva per group over 30 min. Statistical comparisons were determined by one- or two-way ANOVA followed by Dunnett’s (compared with control) or Tukey’s (multiple comparisons) post hoc tests (* *p* < 0.05, **** *p* < 0.0001).

Having characterised the effect of ergolide on the regulation of microglial activation, we investigated whether ergolide may also demonstrate direct neuroprotective effects. To determine a tolerable concentration of ergolide in neurons, N2a and SH-SY5Y cells were exposed to ergolide (1, 3, 5, or 10 µM) for 24 h. CCK-8 analysis revealed that ergolide did not negatively impact the viability of N2a cells, at least not at a concentration of up to 5 µM (Figure 8A). However, exposure to ergolide at both 5 µM (*p* < 0.05) and 10 µM (*p* < 0.0001) significantly reduced the viability of SH-SY5Y cells (Figure 8B). This was accompanied by a concomitant increase in cytotoxicity, which is most evident in the response to 10 µM (*p* < 0.0001; Figure 8C). Having determined 3 µM as a tolerable concentration in both neuronal cells, we assessed the impact of ergolide on oxidative stress-induced neurotoxicity. SH-SY5Y cells were treated with H_2_O_2_ (0.5 mM; 24 h) in the presence and absence of ergolide (3 µM). Incubation with H_2_O_2_ significantly reduced SH-SY5Y cell viability (40.5% ± 6.4%; *p* < 0.0001; Figure 8D) and increased cytotoxicity (151.9% ± 6.7%; *p* < 0.0001; Figure 8E). However, co-incubation with ergolide did not impact H_2_O_2_-induced cell death (Figure 8D,E). To further investigate the potential of ergolide to display antioxidant properties, N2a cells were pre-incubated with ergolide (5 µM) or DMSO for 30 min prior to application of the organic peroxide tBHP (200 µM; 4 h) to induce the production of ROS. Here, we observed that tBHP-induced ROS production was further exacerbated by ergolide (*p* < 0.0001; Figure 8F).

To evaluate the influence of ergolide on neuronal stress in vivo, zebrafish larvae at 4 days post-fertilisation (dpf) were exposed to ergolide (5 µM) or an equivalent volume of DMSO vehicle control for 23.5 h. The GABA_A_ receptor antagonist PTZ (15 mM) [81] was added to each well for a further 30 min to induce seizure-like activity. Hyperactivity in response to PTZ was determined as a profound increase in total distance travelled in 30 min in PTZ-stimulated larvae compared with the controls (*p* < 0.0001). However, this hyperactivity was not altered by pre-treatment with ergolide (Figure 8G,H). Taken together, our findings indicate that the anti-inflammatory properties of ergolide are not accompanied by antioxidant or anti-neurotoxic effects. This would imply that any potential neurotherapeutic effects of ergolide would likely stem from its immunoregulatory capacity.

**Figure 9:**
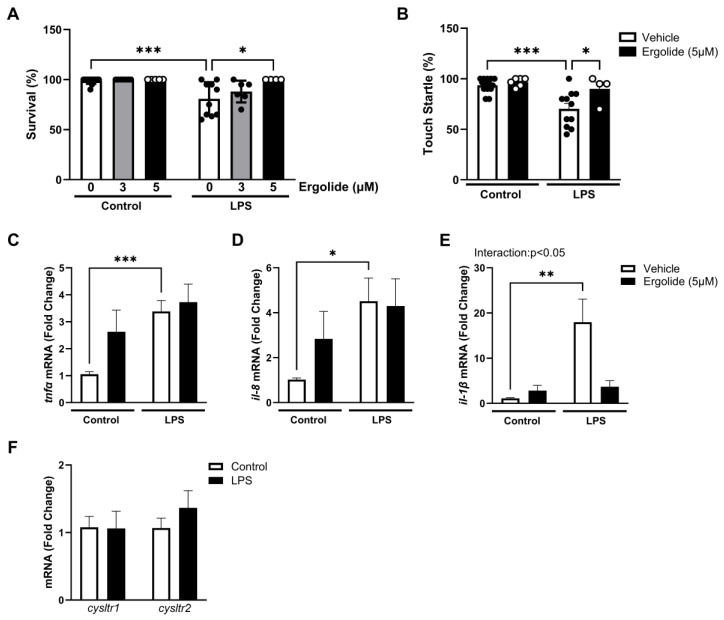
LPS-induced mortality and behavioural dysfunction in zebrafish larvae are restored by ergolide. (**A**) Zebrafish larvae (4 dpf) were exposed to ergolide (3 and 5 μM) in the presence and absence of LPS (50 μg/mL) and larval survival was recorded following 24 h incubation (*n* = 5–12 larvae per group). (**B**) Following 20 h incubation, touch startle response was assessed in groups treated with ergolide (5 μM), with and without LPS *(n* = 6–14 larvae). Data are expressed as a percentage of total larvae exhibiting a positive startle reflex response per treatment group. Dots (overlay) represent data from individual larvae. (**C**–**F**) Larvae at 5 dpf were incubated with ergolide (5 μM) or DMSO (0.025%) for 30 min prior to further application of LPS (50 μg/mL) for 4 h. Expression of *tnfα* (**C**), *il-8* (**D**), *il-1β* (**E**), and *cysltr1* and *cysltr2* (**F**). mRNA was assessed relative to *gapdh* (*n* = 5–13 replicates from 3-6 independent experiments). Data are expressed as mean ± SEM. Statistical comparisons were made using one- or two-way ANOVA, followed by Tukey’s post hoc analysis (* *p* < 0.05, ** *p* < 0.01, and *** *p* < 0.001). Interaction reports findings of two-way ANOVA.

Therefore, to examine the impact of ergolide on inflammatory changes in vivo, zebrafish larvae (4 dpf) were incubated with ergolide (3 and 5 µM) or a vehicle for 24 h in the presence and absence of LPS (50 µg/mL). As zebrafish have been reported to exhibit tolerance to *E. coli*, LPS derived from *P. aeruginosa* was used as a previously characterised model of inflammation [82,83]. Ergolide alone had no effect on the larval survival at either concentration (Figure 9A). Exposure to LPS significantly reduced larval survival compared with untreated controls (*p* < 0.01; Figure 9A). However, when co-incubated with ergolide at 5 µM, the proportion of surviving larvae was significantly higher compared with those exposed to LPS alone (*p* < 0.05; Figure 9A). Exposure to LPS also reduced the touch startle response in the 5 dpf larvae, indicated by a reduction in the proportion of larvae exhibiting a rapid, escape-like reflex movement away from the touch stimulus (*p* < 0.001; Figure 9B). However, this was significantly restored by co-treatment with ergolide (*p* < 0.05). To investigate whether this restorative effect may be mediated through similar regulation of inflammation as seen in microglia, zebrafish larvae (5 dpf) were treated with ergolide ± LPS for 4 h and RNA was isolated to assess inflammatory-associated gene expression. LPS exposure significantly increased the expression of *tnfα* (*p* < 0.001; Figure 9C), *il-8* (*p* < 0.05; Figure 9D), and *il-1β* (*p* < 0.01; Figure 9E) mRNA compared with the controls. While ergolide did not ameliorate the LPS-induced changes in *tnfα* or *il-8*, the upregulation in the expression of *il-1β* was significantly reduced in LPS-stimulated larvae (*p* < 0.05; Figure 9E). Interestingly, however, exposure to ergolide alone led to an increase in *tnfα* and *il-8* expression, which reached significance for *tnfα* (ergolide effect: *p* < 0.001; Figure 9C). Similarly to that seen in microglia, LPS stimulation did not alter the expression of either *cysltr1* and *cysltr2* mRNA compared with the untreated larvae (Figure 9F). Unlike our observation in LTA-stimulated microglia, these findings do not associate an increase in CysLTR expression with LPS-induced sickness behaviour in zebrafish. However, they do emphasise the inflammatory basis of these effects. Moreover, we may hypothesise that the restorative effects of ergolide against LPS-induced toxicity and behavioural dysfunction is likely due to regulation of IL-1β production.

## 3. Discussion

A previous study reported that ergolide exerts anti-inflammatory effects in macrophages by inhibiting TLR-NFκB signalling [58]. Our findings demonstrate similar effects in microglia, suggesting that these effects extend beyond systemic inflammation. These properties infer that ergolide may have therapeutic value in common with other terpenes. For example, D-limonene, naturally found in citrus fruits, modulates the NFκB pathway, and was found to reduce production in a mouse model of ulcerative colitis [84]. Similar effects have been reported in microglia, in which terpenes such as thymoquinone and andrographolide have been shown to suppress NFκB activity in response to Aβ and TLR stimulation [85,86]. We propose that ergolide may offer therapeutic benefit to neuroinflammatory disorders. However, unlike other terpenes, our study highlighted that ergolide lacks antioxidant properties. D-limonene, by contrast, has also been seen to enhance antioxidant enzymes such as catalase and peroxidase in H_2_O_2_-stimulated lymph node cells [87], while monoterpenes 1,8-cineole and camphene inhibit ROS generation and promote the expression of endogenous antioxidant proteins [88,89].

In contrast to these agents, ergolide did not mitigate neuronal damage induced by H_2_O_2_ and tBHP, but moreover, exacerbated ROS production in N2a cells. Notably, the same concentration that produced anti-inflammatory effects in microglia reduced the viability of SH-SY5Y neuroblastoma cells. While monoterpenes such as D-limonene and 1,8-cineole are noted for their antioxidant properties [87,88], sesquiterpenes such as β-caryophyllene and humulene, and sesquiterpene lactones such as alantolactone, can promote ROS generation and induce neuronal death [90,91]. Ergolide has previously been reported to be anti-proliferative in uveal melanoma and acute lymphoblastic leukaemia cells [61,62]. However, this cytotoxicity was selective to cancer cell lines, with no significant effect in non-tumourous cells [61]. Yami and colleagues (2020) further demonstrated that ergolide-induced cytotoxicity is mediated by ROS-dependent late apoptosis [61]. SH-SY5Y cells are a subline of the SK-N-SH cell line derived from a bone marrow biopsy of a neuroblastoma patient [92,93], and differ from immortalised mouse BV2 microglia, which is generated by a recombinant retrovirus [94]. Neuroblastoma is a rare form of cancer most commonly impacting the nervous system of children under the age of 5. Surgical intervention, chemo- and radiation therapy, is the most used combination treatment, but up to 50% of high-risk cases are treatment-resistant [94]. Given the risks associated with these treatments, particularly in young patients, our findings highlight the potential of ergolide as a ROS-mediated cytotoxic agent in neuroblastoma. However, understanding its concentration-dependent cytotoxic effects will be critical for evaluating its therapeutic potential in this context.

Our investigation of the anti-inflammatory mechanism of ergolide in microglia identified the inhibition of NFκB as a key driver. While this has been observed previously in systemic inflammatory models, this is typically associated with suppression of MAP kinase activity [59,60]. In contrast, our findings suggest that ergolide inhibits NFκB in microglia independently of MAP kinases. Although activation of the transcription factor Nrf2 via PPARγ is a common mechanism of plant-derived compounds [73,95], our data show that ergolide suppresses microglial activation despite inhibition of PPARγ, suggesting this as an unlikely mechanism. Activation of the Nrf2 pathway leads to the induction of cytoprotective genes, including hemeoxygenase (HO)-1, which can also inhibit NFκB [96]. Ergolide has previously been shown to induce HO-1 activity [62] similar to other terpenes such as lanostane triterpenes [97] and tussilagone [98], resulting in reduced cytokine production. Although we did not detect HO-1 expression in our samples, the potential involvement of Nrf2 signalling in ergolide’s effects on microglia warrants further investigation. This may also explain the cytotoxic effect of ergolide in neuroblastoma cells, since HO-1 has been implicated in promoting apoptosis of tumour cells [99].

Inhibition of CysLTRs has been shown previously to modulate microglial activation, primarily through NFκB-dependent pathways [39,44]. Our study supports and extends these findings. We demonstrate, for the first time, a role for CysLTR2 in regulating TLR2-mediated microglial activation, implicating the CysLT pathway as a mediator of the anti-inflammatory effects of ergolide. This highlights the therapeutic potential of targeting CysLTRs in diseases such as AD and PD, where TLR2-driven inflammation is prominent. Indeed, the CysLTR1 antagonist pranlukast is shown to mitigate the neuronal dysfunction and cognitive impairment associated with Aβ [46]. Interestingly 6-shogaol, the primary bioactive component of ginger, reduced Aβ burden and restored cognition in the APP/PS1 mouse via CysLTR1 inhibition [100]. Similarly, the CysLTR1 antagonist montelukast was recently found to alleviate asthma-exacerbated cognitive deficits in APP/PS1 mice [101]. Despite these advances, the role of endogenous CysLTs in microglial activation remains minimally explored. The synthetic LTC4 analogue, *N*-methyl Leukotriene C4 (NMLTC4), upregulates NFκB-dependent TNFα and IL-1β production in microglia, an effect reversed by CysLTR2 inhibition [38,39]. Based on this, we hypothesised that LTD4 would elicit similar effects. Interestingly, in BV2 cells, LTD4 selectively increased IL-6 without affecting TNFα or NO. Consistent with this, liver macrophages exposed to 20 nM LTD4 were found not to release TNFα unless co-stimulated with LPS [102], suggesting that LTD4 may amplify, but not independently initiate, an inflammatory response. LTD4 also induced IL-8 secretion in CysLTR1-expressing HEK293 cells in a biphasic and concentration-dependent manner, peaking in response to 0.1 nM [103]. In BV2 cells overexpressing CysLTRs, LTD4 induced IL-1β and phagocytosis via CysLTR2 activation [37]. Our findings add to the limited studies assessing direct LTD4 effects on microglia with endogenous receptor expression. We show that, unlike previous reports of TNFα following 6 h exposure to LTD4 [38], we observed reduced TNFα after 24 h exposure, supporting a potential biphasic role in the microglial inflammatory response.

While we identified a role for CysLTR2 activation in the microglial response to LTA, we did not directly measure endogenous CysLT production, which would further elucidate the role of the pathway in microglial inflammation and in ergolide’s mechanism of action. Camphene has been shown to inhibit 5-lipoxygenase (5LOX), a key enzyme in CysLT biosynthesis, in pancreatic cancer cells [104], and similar effects have been reported with terpenes such as thymoquinone and glycyrrhetinic acids [105,106]. On that basis, we propose that ergolide may also suppress inflammatory responses, at least partly, via inhibition of CysLT production. This suggests that modulation of the CysLT pathway may be a common mechanism of terpene bioactivity. Elevated levels of LTC4 and the non-CysLT LTB4 have been detected in cerebrospinal fluid of MS patients [40], with 5LOX upregulated in a murine model of the disease [41]. Similarly, these pathway components were increased in a rodent model of Gulf War Illness (GWI), characterised by neurological symptoms including chronic fatigue, cognitive dysfunction, and pain. Notably, CysLT upregulation in GWI appears to be brain-specific, with no corresponding change in CysLT peripheral blood inflammatory markers [79]. This further underscores the role of the CysLT pathway in neuroinflammation and neuronal dysfunction, and expands the potential application of ergolide as a neurotherapeutic agent.

Our in vivo analysis revealed that ergolide mitigated some LPS-induced effects in zebrafish larvae. However, unlike our in vitro findings, and those previously reported in BV2 [107] and neuronal cells [100], LPS challenge did not upregulate CysLTR expression in zebrafish larvae. Kyritsis and colleagues (2012) previously reported elevated CysLTs in adult zebrafish following traumatic brain injury, implicating this pathway in neurogenesis post-trauma [108]. As our study assessed the response to systemic LPS application rather than brain-specific insult, the effects of systemic inflammation likely confounded the CNS response. As such, we cannot exclude a role of CysLTRs in the zebrafish CNS, or indeed the impact of ergolide on this pathway. However, the study by Kyritsis et al. (2012) also highlighted key differences in neuroinflammatory processes between zebrafish and mammals, whereby pathways detrimental in mammals may support neuroprotection in zebrafish [108]. CysLTR bioactivity has been previously reported in zebrafish, with CysLT-targeting drugs showing anti-angiogenic [109] and anti-cancer [110] effects in models of ocular disease. To clarify the impact of ergolide on neuronal dysfunction, future studies should explore the CNS-specific expression of CysLTRs and endogenous CysLT production in the zebrafish brain. Moreover, understanding how inflammatory challenge regulates these pathways would enhance opportunities to genetically manipulate their expression and further assess their therapeutic potential in neuroinflammatory disease.

The CysLT pathway has been strongly implicated in the pathogenesis of epilepsy [111]. LTD4 was shown to exacerbate PTZ-induced seizures in mice [112], while montelukast reduced seizures in the same model [113]. The zebrafish PTZ model has become an attractive tool for investigating epilepsy mechanisms and testing novel anticonvulsant therapies [114,115,116], yet the CysLT pathway has not been explored in this model. In our study, ergolide did not mitigate seizure-like activity in PTZ-stimulated larvae, suggesting that it lacks anticonvulsant properties or that CysLTs do not contribute to this pathology. Previous reports propose that CysLT pathway inhibition confers neuroprotection indirectly by modulating microglial function, as antagonism of CysLTR2 on microglia reduced the neurotoxic effects of microglial-conditioned media [38,107]. However, PTZ-induced seizures result from GABA-ergic inhibition, leading to a complex pathology of inflammation, excitotoxicity, oxidative stress, and apoptosis [115,117]. If indeed ergolide acts primarily via the CysLT pathway, this may explain its lack of efficacy against direct neuronal dysfunction in PTZ-stimulated zebrafish compared to its anti-inflammatory effects in LPS models. Since ergolide did not exhibit antioxidant or neuroprotective effects in vitro, agents targeting multiple facets of seizure pathology may be required for anticonvulsant efficacy in this model. We also acknowledge the limitations of the zebrafish model, which, while vertebrate, does not fully recapitulate the complexity of the mammalian CNS [118]. Moreover, although zebrafish express TLR4, they lack several of the accessory proteins essential for canonical LPS signalling in mammalian cells. Our findings align with previous reports of zebrafish responsiveness to LPS [82,119], though some of the inflammatory effects we observed likely occur via TLR4-independent mechanisms [120,121].

Collectively, our findings establish ergolide as a potent anti-inflammatory agent, particularly in the context of neuroinflammation. However, its broader neurotherapeutic potential requires further investigation. While ergolide mitigated the toxicity and behavioural dysfunction associated with inflammatory challenge, our data do not support its use in excitotoxic neuronal damage, such as epilepsy. Given that terpenoids, including some sesquiterpene lactones, modulate ROS production in a biphasic, concentration-dependent manner [90,91,122], ergolide may exert antioxidant and anticonvulsant effects at higher doses. As we did not assess the dose-dependence of ergolide’s effects in vivo, and considering the limitations of our zebrafish model discussed above, the current study cannot fully evaluate its therapeutic efficacy. Nonetheless, our data provide compelling evidence to support the further exploration of ergolide as a candidate therapeutic for inflammatory-associated conditions, including neurodegenerative disease.

## 4. Materials and Methods

### 4.1. Maintenance and Treatment of BV2, N9, and SH-SY5Y Cells

Murine BV2 and N9 cells were used as well-defined in vitro models with which to assess microglial activation in response to TLR stimuli [8,69,70]. Murine N2a and human SH-SY5Y neuroblastoma cell lines were used as models of neurons. Cells were grown in 75 cm^2^ flasks containing Dulbecco’s modified Eagle’s medium Hams F-12 50/50 mix with L-glutamine (DMEM/F-12; Corning or Lonza, Slough, UK), which was further supplemented with heat-inactivated foetal bovine serum (FBS; 10%, Gibco, London, UK) and penicillin-streptomycin (100 U/mL; Gibco, UK); referred to as complete DMEM. Cells were maintained in a sterile, humidified environment (37 °C; 5% CO_2_) until confluent. Adherent BV2 and N9 and N2a cells were removed from flasks using a cell scraper (Sarstedt, Leicester, UK). SH-SY5Y cells were rinsed with Dulbecco’s Phosphate Buffered Saline (DPBS; Corning, Deeside, UK), incubated with 0.05% Trypsin-EDTA (Gibco, UK) for 3–5 min, and neutralised with complete DMEM. Cell suspensions were centrifuged at 2000 rpm for 3 min and counted using a Neubauer-improved haemocytometer. Cells were seeded in 24-well (1.5 × 10^5^ cells/well) or 96-well (1 × 10^4^ cells/well) culture plates and incubated overnight at 37 °C.

To induce microglial activation, BV2 and N9 cells in 24-well plates were stimulated with TLR agonists, lipoteichoic acid (LTA derived from *S. aureus*; 5 µg/mL; Sigma-Aldrich, Gillingham, UK), or lipopolysaccharide (LPS from *E. coli*; 5 ng/mL or 200 ng/mL; Enzo Life Sciences, Exeter, UK) as previously described [7,8,71,123]. Cells were co-incubated with the ergolide (0–10 µM; MedChemExpress, Monmouth Junction, NJ, USA) or an equivalent volume of dimethylsulfoxide (DMSO; Sigma-Aldrich, UK) as a vehicle control. In a separate experiment, BV2 cells were incubated with LTA (5 µg/mL) and ergolide (5 µM) in the presence and absence of the PPARγ antagonist GW9662 (10 µM; MedChemExpress, Sollentuna, Sweden, CysLTR1 antagonist montelukast (AOBIUS, Gloucester, MA, USA), or CysLTR2 antagonist HAMI3379 (0.1, 1, and 10 µM; APExBIO, Houston, TX, USA) for 24 h. BV2 cells were stimulated with the CysLT, LTD4 (0.01, 0.1, and 1 µM) in the presence and absence of ergolide (5 µM).

N2a and SH-SY5Y cells were exposed to oxidative compounds tert-butyl hydroperoxide (tBHP; 200 µM) or H_2_O_2_ (0.5 mM) for either 4 or 24 h, respectively. Cells were co-incubated with ergolide (N: 1, 3, and 5 µM; SH-SY5Y: 1, 3, 5, and 10 µM). Treatments for all cells were carried out in duplicate or triplicate in a final volume of 100 µL or 200 µL per well. Data were acquired based on results from a minimum of 3 independent experiments, with 2 or 3 treatment replicates per experiment.

### 4.2. NFκB Luciferase Assay

THP1 NFκB Lucia™ cells stably express a luciferase reporter gene controlled by NFκB. Cells were maintained in Roswell Park Memorial Institute 1640 media (RPMI; Gibco, UK) containing D-glucose (4.5 g/L), L-glutamine, sodium pyruvate (110 mg/L), and sodium bicarbonate (1.5 g/L) and further supplemented with heat-inactivated FBS (10% *v*/*v*), penicillin-streptomycin (100 U/mL), and normacin (100 µg/mL; Invitrogen, Paisley, UK). The cells were maintained in a sterile, humidified environment (37 °C; 5% CO_2_) in 75 cm^2^ flasks, and regularly counted using a haemocytometer to sustain a density not exceeding 1 × 10^6^ cells/mL. For antibiotic selection of the transfected cells, zeocin (Invitrogen, UK) was added in alternating concentrations of 100 µg/mL or 10 µg/mL every two weeks. Cells were seeded in a 96-well plate (1 × 10^5^ cells/well) and treated with LTA (5 µg/ml) or LPS (5 ng/mL) in the presence and absence of ergolide (5 µM) for 24 h at 37 °C. Following treatment, plates were centrifuged as above, cell supernatant was removed to a white opaque 96-well plate, and QUANTI-Luc™ Luciferase Reagent (Invitrogen, UK) was added. Luminescence readings were performed using the CLARIOstar Plus microplate reader (BMG Labtech, Ortenberg, Germany) set for emission detection in the range of 465–493 nm. Radioimmunoprecipitation assay (RIPA) buffer (containing: 50 mM Tris-HCl, 150 mM NaCl, 1% *v*/*v* IGEPAL^®^, 0.5% *w*/*v* Sodium deoxycholate, 0.1% *w*/*v* SDS) was applied to THP1 cells and protein concentration was determined for each corresponding well using a BCA assay (ThermoFisher, Loughborough, UK) as previously described [8]. All luciferase readings were normalised to their corresponding protein content per well and data were expressed as a proportion of the control group.

### 4.3. Cell Viability and Cytotoxicity Analysis

Cell viability was determined using the Cell Counting Kit-8 (CCK-8; Dojindo Laboratories, Rockville, MD, USA), in accordance with manufacturers guidelines. In brief, supernatant was removed from BV2 and SH-SY5Y cells following treatment, replaced with complete DMEM containing 10% CCK-8 reagent (100 µL/well), and incubated for 1–2 h (37 °C; 5% CO_2_). In total, 50 µL of supernatant was moved to a new 96-well plate and absorbance was measured at 450 nm. Cytotoxicity was determined using the lactate dehydrogenase (LDH) assay (CyQUANT™ LDH Cytotoxicity Assay; Invitrogen, UK) according to manufacturer’s instructions. Following cell treatment, supernatant (50 µL) was moved to a new 96-well plate, along with a known concentration of LDH as a positive control for the assay and complete DMEM as a blank. An equal volume of the reaction buffer was added to each sample, and the plate was incubated on an orbital shaker for 30 min at room temperature. The reaction was stopped by application of assay stop solution. Absorbances were read at 490 nm and 680 nm. Cytotoxicity was calculated as a percentage of the control group. Absorbance values were measured using the SpectraMax M3 microplate spectrophotometer and SoftMax Pro 6.2.1 software (Molecular Devices, San Jose, CA, USA). Cell viability and cytotoxicity were calculated as a percentage of the control group.

### 4.4. Reactive Oxygen Species (ROS) Analysis

N2a cells were treated with ergolide (5 µM) prior to co-application of tBHP (200 µM) for a further 4 h. Cells were rinsed with warm DPBS, and 2′,7′-dichlorofluorescein diacetate (DCFDA; 20 mM; Sigma-Aldrich, UK) was applied and incubated for 1 h at 37 °C. Cell supernatant was removed and cells were lysed in RIPA buffer (100 µL/well) in the dark and on ice for 30 min. In total, 50 µL of each lysate was transferred to the corresponding wells of a black 96-well plate and fluorescence was measured at an excitation wavelength of 488 nm and emission of 535 nm using a SpectraMax M3 microplate spectrophotometer (Molecular Devices, USA). Finally, 25 µL of each remaining sample was used for determination of protein concentration using a BCA assay.

### 4.5. Analysis of Cytokine and Nitrite Concentration

Supernatant concentration of TNFα, IL-6, and MCP1 was determined by enzyme-linked immunosorbent assay (ELISA), carried out according to the manufacturer’s guidelines (Invitrogen and Biolegend, London, UK) and as previously described [8]. Cytokine concentration was determined against standard curves, prepared using the recombinant TNFα, IL-6, or MCP1 provided. Absorbance was read at 450 nm. Nitrite was measured in cell supernatants using the colorimetric Griess assay as previously described [7,123]. In brief, 50 µL of supernatant sample was applied in duplicate to each well of a 96-well plate and incubated with an equal volume of modified Griess reagent (Sigma-Aldrich, UK) for 20 min at room temperature. Absorbance was measured at 540 nm. Absorbance values were determined using a SpectraMax M3 microplate spectrophotometer. Due to variability in basal expression between independent cell cultures, data are expressed as a proportion of the control group within each experiment.

### 4.6. Western Immunoblot Analysis

Expression of phosphorylated MAP kinases was determined by Western immunoblot, according to a previously described protocol [8]. Briefly, BV2 cells were lysed in RIPA buffer supplemented with protease and phosphatase inhibitor cocktails (Sigma-Aldrich, UK). Total protein concentration was quantified and equalised based on a BCA assay, diluted with Laemmli sample buffer (6×; 15% SDS, 15% β-mercaptoethanol, 50% glycerol, 0.01% bromophenol blue, and 0.125 mM Tris; Sigma-Aldrich, UK), and protein was denatured at 77 °C. Samples were separated using SDS-PAGE, transferred to nitrocellulose membranes, and blocked in 5% semi-skimmed milk (Sigma-Aldrich, UK) prior to overnight incubation with antibodies against p-JNK (Thr183/Tyr185), p-p38 (Thr180/Tyr182) (1:500–1:1000; Cell Signaling, Boston, MA, USA), and GAPDH (1:10,000; Millipore, Watford, UK) at 4 °C. Membranes were washed and incubated with anti-mouse or anti-rabbit HRP antibodies (Jackson Laboratories, London, UK) for 1 h. Immunoreactive bands were visualised with SuperSignal™ West Pico PLUS substrate (ThermoFisher, UK) using the Vilber Fusion FX Chemiluminescent Imager and analysed with ImageJ software (Ver. 1.32n).

### 4.7. Analysis of Gene Expression

RNA was extracted from BV2 cells using E.Z.N.A.^®^ Total RNA Kit 1 (Omega Bio-tek, London, UK) in accordance with manufacturer’s instructions, or from whole zebrafish larvae (10 larvae pooled per treatment replicate) via standard TRIzol^®^ (Invitrogen, UK) extraction methods. cDNA was synthesised using the SuperScript™ III Reverse Transcriptase System (Invitrogen, UK) and stored at −20 °C. Expression of *il-1β*, *tnfα*, *il-8 cysltr1*, *cysltr2,* and *gapdh* mRNA (zebrafish), and *Cysltr1*, *Cysltr2*, and *Gapdh* mRNA (BV2) was analysed using quantitative real-time PCR performed on a QuantStudio 7Flex qPCR System (Applied Biosciences, London, UK). Each reaction used SYBR™ Green PCR Master Mix (Applied Biosciences, London, UK) and primers for equivalent mouse (KiCqStart; Sigma-Aldrich, UK) or zebrafish (Eurofins, Hamburg, Germany) genes (see Table 2). The quantification cycle (Ct) values were determined for each sample, and relative gene expression levels were calculated using the comparative Ct (ΔΔCt) method.

### 4.8. Zebrafish Maintenance

All zebrafish experiments were conducted on larvae up to a maximum of 5 days post-fertilisation (dpf) in accordance with European Directive 2010/63/EU, and with ethical exemption from the UCD Animal Research Ethics Committee (AREC-E-19-35-Costello). Adult Tübingen (WT-Tü) zebrafish from in-house colonies were used to generate larvae through natural spawning. Embryos were maintained in 90 mm Petri dishes containing embryo medium (0.137 M NaCl, 5.4 mM KCl, 5.5 mM Na_2_HPO_4_, 0.44 mM KH_2_PO_4_, 1.3 mM CaCl_2_, 1.0 mM MgSO_4_, and 4.2 mM NaHCO_3_, containing methylene blue and with conductivity ~1500 μS, pH 7.2) and maintained at 27 °C on a 14 h/10 h light–dark cycle.

### 4.9. LPS Stimulation in Zebrafish Larvae

To assess the anti-inflammatory effect of ergolide in LPS-stimulated zebrafish, larvae (4 dpf) were placed in a 24-well plate (5–10 larvae per well). Larvae were pretreated in embryo medium containing ergolide (3 or 5 µM) or an equivalent volume of DMSO (0.025%) for 30 min, and LPS (derived from *P. aeruginosa*, Sigma-Aldrich, UK) was added to a subset of wells to achieve a final concentration of 50 µg/mL. The plate was incubated at 27 °C for 20 h prior to carrying out touch startle analysis as below. Following a further 4 h incubation, larvae were harvested for RNA extraction as described above.

### 4.10. Survival and Touch Startle Response Assay

Groups of 5 or 10 larvae (4 dpf) were placed in individual wells of a 24-well plate with control embryo media or media containing the relevant drug. Each treatment was applied to 2–4 replicate wells. Larvae lacking a detectable heartbeat after 24 h treatment were recorded as deceased. The percentage of surviving larvae per treatment group per plate was subsequently recorded. Following treatments ranging from 3 to 20 h, larvae were startled by light mechanical stimulation to the tail using the tip of a dissection teasing needle. A positive startle response was recorded as a characteristic escape-like movement or darting motion away from the stimulus. Assessment of startle was carried out on all larvae prior to determination of survival. Therefore, deceased larvae were recorded as non-responsive. The percentage of startling larvae per treatment group per plate was recorded.

### 4.11. Assessment of PTZ-Induced Hyperactivity

Individual larvae (4 dpf) were placed in each well of a 48-well plate (one larva per well) with embryo media containing ergolide (5 μM) or a vehicle (0.025% DMSO) for 23.5 h. Following this, a subset of larvae received PTZ at a final concentration of 15 mM or the equivalent volume of control media. The plate was then placed in the Zebrabox VideoTrack System for Zebrafish™ (ViewPoint Life Sciences, Lyon, France) and the larvae were allowed to acclimatise to the system for 5 min. Locomotor activity per second was monitored over a 30 min period and tracked using ViewPoint software v5.33.0.260 (ZebraLab, ViewPoint Life Sciences, Lyon, France). Total distance travelled was recorded within 5 min blocks during the 30 min experiment.

### 4.12. Statistical Analysis

Statistical comparisons were made using two-way analysis of variance (ANOVA) to assess the effects and interactions between two independent variables, followed by Tukey’s post hoc analysis. One-way ANOVA was used to compare multiple variables, followed by Dunnet’s analysis to determine differences with respect to the control group. Corrections were applied for all multiple comparison analyses. All graphs and statistical analysis were carried out using GraphPad Prism 10 software. Statistical significance is represented as * *p* < 0.05, ** *p* < 0.01, *** *p* < 0.001, and *** *p* < 0.0001.

## Figures and Tables

**Table 1:** Supernatant concentration of inflammatory mediators from BV2 microglia stimulated with LTA (5 µg/mL) or LPS (5 ng/mL) in the presence and absence of erolide (5 µM; 24 h). * *p* < 0.05, ** *p* < 0.01, *** *p* < 0.001, and two-way ANOVA ergolide effect; *n* = 9 replicates from 3 independent experiments.

	LTA (Mean ± SEM)	LTA + Ergolide (Mean ± SEM)	*p*	LPS (Mean ± SEM)	LPS + Ergolide (Mean ± SEM)	*p*
Nitrite (µM)	21.86 ± 3.89	9.80 ± 0.96	*	19.24 ± 1.32	11.54 ± 1.66	***
TNFα (pg/mL)	145.21 ± 21.96	102.73 ± 23.63	*-*	155.07 ± 18.72	101.05 ± 15.6	*
IL-6 (pg/mL)	9005.73 ± 867.27	5245.29 ± 1244.85	*	8894.52 ± 1039.8	5505.03 ± 795.16	*
MCP1 (pg/mL)	2102.51 ± 173.55	1659.62 ± 131.09	**	2244.45 ± 189.88	1680.56 ± 176.14	**

**Table 2:** Primer sequences for *Danio rerio* and mouse genes.

Species	Gene	Forward Primer (5′->3′)	Reverse Primer (5′->3′)
*danio rerio*	*cysltr1*	ACTTTTTGTGAGTGGAAATGTGTT	ACCACTCATGGAGGTCCTGTT
*danio rerio*	*cysltr2*	TGTTTGGAGCTCGCACATGA	ATGATCACCAAAGCGCAAGC
*danio rerio*	*gapdh*	AAGTCAGTGGACACAACCTGG	CAAGAAAGTCGTCAAGGCTGC
*danio rerio*	*il1* *β*	TGTGTTATTGTTTTCCTGGCATTTC	GCGACAGCGTGGATCTACAG
*danio rerio*	*il8*	CACAAAGGCTGCCATTCACT	GATTGATGGTGTGGCTCAGGT
*danio rerio*	*tnf* *α*	CACAAAGGCTGCCATTCACT	GATTGATGGTGTGGCTCAGGT
*mouse*	*Cysltr1*	AAACTGTAAGCTTGGAATC	TACATTTCTGCAATCACAGC
*mouse*	*Cysltr2*	TCTGTTAGAGCCACTTGTAG	CACATGATGGACATCTTATCTC
*mouse*	*Gapdh*	CTAATGACCACAGTCCATTC	GTGGGATGATGTTTTGGTG

## Data Availability

Data are contained within the article.

## References

[1] Bartman S., Coppotelli G., Ross J.M. (2024). Mitochondrial Dysfunction: A Key Player in Brain Aging and Diseases. Curr. Issues Mol. Biol..

[2] Zhang W., Sun H.S., Wang X., Dumont A.S., Liu Q. (2024). Cellular senescence, DNA damage, and neuroinflammation in the aging brain. Trends Neurosci..

[3] Golde T.E. (2022). Disease-Modifying Therapies for Alzheimer’s Disease: More Questions than Answers. Neurotherapeutics.

[4] Yi L.X., Tan E.K., Zhou Z.D. (2024). Passive immunotherapy for Alzheimer’s disease: Challenges & future directions. J. Transl. Med..

[5] Hanke M.L., Kielian T. (2011). Toll-like receptors in health and disease in the brain: Mechanisms and therapeutic potential. Clin. Sci..

[6] Dabi Y.T., Ajagbe A.O., Degechisa S.T. (2023). Toll-like receptors in pathogenesis of neurodegenerative diseases and their therapeutic potential. Immun. Inflamm. Dis..

[7] Howe A.M., Burke S., O’Reilly M.E., McGillicuddy F.C., Costello D.A. (2022). Palmitic Acid and Oleic Acid Differently Modulate TLR2-Mediated Inflammatory Responses in Microglia and Macrophages. Mol. Neurobiol..

[8] Howe A.M., Cosgrave A., O’Murchu M., Britchfield C., Mulvagh A., Fernandez-Perez I., Dykstra M., Jones A.C., Costello D.A. (2020). Characterising lipoteichoic acid as an in vitro model of acute neuroinflammation. Int. Immunopharmacol..

[9] Costello D.A., Lyons A., Denieffe S., Browne T.C., Cox F.F., Lynch M.A. (2011). Long term potentiation is impaired in membrane glycoprotein CD200-deficient mice: A role for Toll-like receptor activation. J. Biol. Chem..

[10] Costello D.A., Lynch M.A. (2013). Toll-like receptor 3 activation modulates hippocampal network excitability, via glial production of interferon-β. Hippocampus.

[11] Costello D.A., Carney D.G., Lynch M.A. (2015). α-TLR2 antibody attenuates the Aβ-mediated inflammatory response in microglia through enhanced expression of SIGIRR. Brain Behav. Immun..

[12] Liu S., Liu Y., Hao W., Wolf L., Kiliaan A.J., Penke B., Rube C.E., Walter J., Heneka M.T., Hartmann T. (2012). TLR2 is a primary receptor for Alzheimer’s amyloid β peptide to trigger neuroinflammatory activation. J. Immunol..

[13] Richard K.L., Filali M., Prefontaine P., Rivest S. (2008). Toll-like receptor 2 acts as a natural innate immune receptor to clear amyloid β 1-42 and delay the cognitive decline in a mouse model of Alzheimer’s disease. J. Neurosci..

[14] Kim C., Ho D.H., Suk J.E., You S., Michael S., Kang J., Joong Lee S., Masliah E., Hwang D., Lee H.J. (2013). Neuron-released oligomeric α-synuclein is an endogenous agonist of TLR2 for paracrine activation of microglia. Nat. Commun..

[15] Soraci L., Gambuzza M.E., Biscetti L., Lagana P., Lo Russo C., Buda A., Barresi G., Corsonello A., Lattanzio F., Lorello G. (2023). Toll-like receptors and NLRP3 inflammasome-dependent pathways in Parkinson’s disease: Mechanisms and therapeutic implications. J. Neurol..

[16] Heneka M.T. (2017). Inflammasome activation and innate immunity in Alzheimer’s disease. Brain Pathol..

[17] Sivamaruthi B.S., Raghani N., Chorawala M., Bhattacharya S., Prajapati B.G., Elossaily G.M., Chaiyasut C. (2023). NF-κB Pathway and Its Inhibitors: A Promising Frontier in the Management of Alzheimer’s Disease. Biomedicines.

[18] Valera E., Mante M., Anderson S., Rockenstein E., Masliah E. (2015). Lenalidomide reduces microglial activation and behavioral deficits in a transgenic model of Parkinson’s disease. J. Neuroinflamm..

[19] Kong F., Jiang X., Wang R., Zhai S., Zhang Y., Wang D. (2020). Forsythoside B attenuates memory impairment and neuroinflammation via inhibition on NF-κB signaling in Alzheimer’s disease. J. Neuroinflamm..

[20] Li Y., Xia Y., Yin S., Wan F., Hu J., Kou L., Sun Y., Wu J., Zhou Q., Huang J. (2021). Targeting Microglial α-Synuclein/TLRs/NF-κB/NLRP3 Inflammasome Axis in Parkinson’s Disease. Front. Immunol..

[21] Kacimi R., Giffard R.G., Yenari M.A. (2011). Endotoxin-activated microglia injure brain derived endothelial cells via NF-κB, JAK-STAT and JNK stress kinase pathways. J. Inflamm..

[22] Yu Y., Shen Q., Lai Y., Park S.Y., Ou X., Lin D., Jin M., Zhang W. (2018). Anti-inflammatory Effects of Curcumin in Microglial Cells. Front. Pharmacol..

[23] Hensley K., Floyd R.A., Zheng N.Y., Nael R., Robinson K.A., Nguyen X., Pye Q.N., Stewart C.A., Geddes J., Markesbery W.R. (1999). p38 kinase is activated in the Alzheimer’s disease brain. J. Neurochem..

[24] Kheiri G., Dolatshahi M., Rahmani F., Rezaei N. (2018). Role of p38/MAPKs in Alzheimer’s disease: Implications for amyloid β toxicity targeted therapy. Rev. Neurosci..

[25] Ashabi G., Ramin M., Azizi P., Taslimi Z., Alamdary S.Z., Haghparast A., Ansari N., Motamedi F., Khodagholi F. (2012). ERK and p38 inhibitors attenuate memory deficits and increase CREB phosphorylation and PGC-1α levels in Aβ-injected rats. Behav. Brain Res..

[26] Karpenko M.N., Vasilishina A.A., Gromova E.A., Muruzheva Z.M., Miliukhina I.V., Bernadotte A. (2018). Interleukin-1β, interleukin-1 receptor antagonist, interleukin-6, interleukin-10, and tumor necrosis factor-α levels in CSF and serum in relation to the clinical diversity of Parkinson’s disease. Cell Immunol..

[27] Lerche S., Zimmermann M., Wurster I., Roeben B., Fries F.L., Deuschle C., Waniek K., Lachmann I., Gasser T., Jakobi M. (2022). CSF and Serum Levels of Inflammatory Markers in PD: Sparse Correlation, Sex Differences and Association With Neurodegenerative Biomarkers. Front. Neurol..

[28] Llano D.A., Li J., Waring J.F., Ellis T., Devanarayan V., Witte D.G., Lenz R.A. (2012). Cerebrospinal fluid cytokine dynamics differ between Alzheimer disease patients and elderly controls. Alzheimer Dis. Assoc. Disord..

[29] Wang W.Y., Tan M.S., Yu J.T., Tan L. (2015). Role of pro-inflammatory cytokines released from microglia in Alzheimer’s disease. Ann. Transl. Med..

[30] Schuitemaker A., Dik M.G., Veerhuis R., Scheltens P., Schoonenboom N.S., Hack C.E., Blankenstein M.A., Jonker C. (2009). Inflammatory markers in AD and MCI patients with different biomarker profiles. Neurobiol. Aging.

[31] Duro M.V., Ebright B., Yassine H.N. (2022). Lipids and brain inflammation in APOE4-associated dementia. Curr. Opin. Lipidol..

[32] Guan P.P., Liang Y.Y., Cao L.L., Yu X., Wang P. (2019). Cyclooxygenase-2 Induced the β-Amyloid Protein Deposition and Neuronal Apoptosis Via Upregulating the Synthesis of Prostaglandin E_2_ and 15-Deoxy-Δ^12,14^-prostaglandin J_2_. Neurotherapeutics.

[33] Ikeda-Matsuo Y., Miyata H., Mizoguchi T., Ohama E., Naito Y., Uematsu S., Akira S., Sasaki Y., Tanabe M. (2019). Microsomal prostaglandin E synthase-1 is a critical factor in dopaminergic neurodegeneration in Parkinson’s disease. Neurobiol. Dis..

[34] Currais A., Quehenberger O., Armando A.M., Daugherty D., Maher P., Schubert D. (2016). Amyloid proteotoxicity initiates an inflammatory response blocked by cannabinoids. NPJ Aging Mech. Dis..

[35] Busse W.W. (1998). Leukotrienes and inflammation. Am. J. Respir. Crit. Care Med..

[36] Gelosa P., Colazzo F., Tremoli E., Sironi L., Castiglioni L. (2017). Cysteinyl Leukotrienes as Potential Pharmacological Targets for Cerebral Diseases. Mediat. Inflamm..

[37] Yu S.Y., Zhang X.Y., Wang X.R., Xu D.M., Chen L., Zhang L.H., Fang S.H., Lu Y.B., Zhang W.P., Wei E.Q. (2014). Cysteinyl leukotriene receptor 1 mediates LTD4-induced activation of mouse microglial cells in vitro. Acta Pharmacol. Sin..

[38] Zhang X.Y., Wang X.R., Xu D.M., Yu S.Y., Shi Q.J., Zhang L.H., Chen L., Fang S.H., Lu Y.B., Zhang W.P. (2013). HAMI 3379, a CysLT2 receptor antagonist, attenuates ischemia-like neuronal injury by inhibiting microglial activation. J. Pharmacol. Exp. Ther..

[39] Zhao R., Ying M., Gu S., Yin W., Li Y., Yuan H., Fang S., Li M. (2019). Cysteinyl Leukotriene Receptor 2 is Involved in Inflammation and Neuronal Damage by Mediating Microglia M1/M2 Polarization through NF-κB Pathway. Neuroscience.

[40] Neu I., Mallinger J., Wildfeuer A., Mehlber L. (1992). Leukotrienes in the cerebrospinal fluid of multiple sclerosis patients. Acta Neurol. Scand..

[41] Whitney L.W., Ludwin S.K., McFarland H.F., Biddison W.E. (2001). Microarray analysis of gene expression in multiple sclerosis and EAE identifies 5-lipoxygenase as a component of inflammatory lesions. J. Neuroimmunol..

[42] Wang L., Du C., Lv J., Wei W., Cui Y., Xie X. (2011). Antiasthmatic drugs targeting the cysteinyl leukotriene receptor 1 alleviate central nervous system inflammatory cell infiltration and pathogenesis of experimental autoimmune encephalomyelitis. J. Immunol..

[43] Zhang C.T., Lin J.R., Wu F., Ghosh A., Tang S.S., Hu M., Long Y., Sun H.B., Hong H. (2016). Montelukast ameliorates streptozotocin-induced cognitive impairment and neurotoxicity in mice. Neurotoxicology.

[44] Ghosh A., Chen F., Wu F., Tang S.S., Hu M., Long Y., Sun H.B., Kong L.Y., Hong H. (2017). CysLT_1_R downregulation reverses ntracerebroventricular streptozotocin-induced memory impairment via modulation of neuroinflammation in mice. Prog. Neuropsychopharmacol. Biol. Psychiatry.

[45] Cevik B., Solmaz V., Aksoy D., Erbas O. (2015). Montelukast inhibits pentylenetetrazol-induced seizures in rats. Med. Sci. Monit..

[46] Tang S.S., Hong H., Chen L., Mei Z.L., Ji M.J., Xiang G.Q., Li N., Ji H. (2014). Involvement of cysteinyl leukotriene receptor 1 in Aβ1-42-induced neurotoxicity in vitro and in vivo. Neurobiol. Aging.

[47] Tang S.S., Wang X.Y., Hong H., Long Y., Li Y.Q., Xiang G.Q., Jiang L.Y., Zhang H.T., Liu L.P., Miao M.X. (2013). Leukotriene D4 induces cognitive impairment through enhancement of CysLT_1_ R-mediated amyloid-β generation in mice. Neuropharmacology.

[48] Fang S.C., Wang J.J., Chen F., Tang S.S., Mu R.H., Yuan D.H., Zhao J.J., Hong H., Long Y. (2021). Hippocampal CysLT1R overexpression or activation accelerates memory deficits, synaptic dysfunction, and amyloidogenesis in young APP/PS1 transgenic mice. Ann. Transl. Med..

[49] Samanta S., Chakraborty S., Bagchi D. (2024). Pathogenesis of Neurodegenerative Diseases and the Protective Role of Natural Bioactive Components. J. Am. Nutr. Assoc..

[50] An J., Chen B., Kang X., Zhang R., Guo Y., Zhao J., Yang H. (2020). Neuroprotective effects of natural compounds on LPS-induced inflammatory responses in microglia. Am. J. Transl. Res..

[51] Park H.Y., Park C., Hwang H.J., Kim B.W., Kim G.Y., Kim C.M., Kim N.D., Choi Y.H. (2014). 7,8-Dihydroxyflavone attenuates the release of pro-inflammatory mediators and cytokines in lipopolysaccharide-stimulated BV2 microglial cells through the suppression of the NF-κB and MAPK signaling pathways. Int. J. Mol. Med..

[52] Chen M., Chang Y.Y., Huang S., Xiao L.H., Zhou W., Zhang L.Y., Li C., Zhou R.P., Tang J., Lin L. (2018). Aromatic-Turmerone Attenuates LPS-Induced Neuroinflammation and Consequent Memory Impairment by Targeting TLR4-Dependent Signaling Pathway. Mol. Nutr. Food Res..

[53] Hori Y., Tsutsumi R., Nasu K., Boateng A., Ashikari Y., Sugiura M., Nakajima M., Kurauchi Y., Hisatsune A., Katsuki H. (2021). Aromatic-Turmerone Analogs Protect Dopaminergic Neurons in Midbrain Slice Cultures through Their Neuroprotective Activities. Cells.

[54] Mazzantini C., El Bourji Z., Parisio C., Davolio P.L., Cocchi A., Pellegrini-Giampietro D.E., Landucci E. (2024). Anti-Inflammatory Properties of Cannabidiol and Β-Caryophyllene Alone or Combined in an In Vitro Inflammation Model. Pharmaceuticals.

[55] Askari V.R., Shafiee-Nick R. (2019). The protective effects of β-caryophyllene on LPS-induced primary microglia M_1_/M_2_ imbalance: A mechanistic evaluation. Life Sci..

[56] Vieira A.J., Beserra F.P., Souza M.C., Totti B.M., Rozza A.L. (2018). Limonene: Aroma of innovation in health and disease. Chem. Biol. Interact..

[57] Rolnik A., Olas B. (2021). The Plants of the Asteraceae Family as Agents in the Protection of Human Health. Int. J. Mol. Sci..

[58] Han J.W., Lee B.G., Kim Y.K., Yoon J.W., Jin H.K., Hong S., Lee H.Y., Lee K.R., Lee H.W. (2001). Ergolide, sesquiterpene lactone from Inula britannica, inhibits inducible nitric oxide synthase and cyclo-oxygenase-2 expression in RAW 264.7 macrophages through the inactivation of NF-κB. Br. J. Pharmacol..

[59] Song Y.J., Lee D.Y., Kim S.N., Lee K.R., Lee H.W., Han J.W., Kang D.W., Lee H.Y., Kim Y.K. (2005). Apoptotic potential of sesquiterpene lactone ergolide through the inhibition of NF-κB signaling pathway. J. Pharm. Pharmacol..

[60] Meng X., Sun L., Meng X., Bi Q. (2024). The protective effect of Ergolide in osteoarthritis: In vitro and in vivo studies. Int. Immunopharmacol..

[61] Yami A., Hamzeloo-Moghadam M., Darbandi A., Karami A., Mashati P., Takhviji V., Gharehbaghian A. (2020). Ergolide, a potent sesquiterpene lactone induces cell cycle arrest along with ROS-dependent apoptosis and potentiates vincristine cytotoxicity in ALL cell lines. J. Ethnopharmacol..

[62] Sundaramurthi H., Tonelotto V., Wynne K., O’Connell F., O’Reilly E., Costa-Garcia M., Kovacshazi C., Kittel A., Marcone S., Blanco A. (2023). Ergolide mediates anti-cancer effects on metastatic uveal melanoma cells and modulates their cellular and extracellular vesicle proteomes. Open Res. Eur..

[63] Berghmans S., Hunt J., Roach A., Goldsmith P. (2007). Zebrafish offer the potential for a primary screen to identify a wide variety of potential anticonvulsants. Epilepsy Res..

[64] Bashirzade A.A., Zabegalov K.N., Volgin A.D., Belova A.S., Demin K.A., de Abreu M.S., Babchenko V.Y., Bashirzade K.A., Yenkoyan K.B., Tikhonova M.A. (2022). Modeling neurodegenerative disorders in zebrafish. Neurosci. Biobehav. Rev..

[65] Razali K., Othman N., Mohd Nasir M.H., Doolaanea A.A., Kumar J., Ibrahim W.N., Mohamed Ibrahim N., Mohamed W.M.Y. (2021). The Promise of the Zebrafish Model for Parkinson’s Disease: Today’s Science and Tomorrow’s Treatment. Front. Genet..

[66] Liu Y. (2023). Zebrafish as a Model Organism for Studying Pathologic Mechanisms of Neurodegenerative Diseases and other Neural Disorders. Cell Mol. Neurobiol..

[67] Xie Y., Meijer A.H., Schaaf M.J.M. (2020). Modeling Inflammation in Zebrafish for the Development of Anti-inflammatory Drugs. Front. Cell Dev. Biol..

[68] Khan K.M., Collier A.D., Meshalkina D.A., Kysil E.V., Khatsko S.L., Kolesnikova T., Morzherin Y.Y., Warnick J.E., Kalueff A.V., Echevarria D.J. (2017). Zebrafish models in neuropsychopharmacology and CNS drug discovery. Br. J. Pharmacol..

[69] Henn A., Lund S., Hedtjarn M., Schrattenholz A., Porzgen P., Leist M. (2009). The suitability of BV2 cells as alternative model system for primary microglia cultures or for animal experiments examining brain inflammation. ALTEX.

[70] Jiang S., Wan Q., Wang X., Di L., Li X., Kang R., Li S., Huang L. (2023). LXA4 attenuates perioperative neurocognitive disorders by suppressing neuroinflammation and oxidative stress. Int. Immunopharmacol..

[71] Al-Thani N.A., Zinck D., Stewart G.S., Costello D.A. (2024). Modulation of Urea Transport Attenuates TLR2-Mediated Microglial Activation and Upregulates Microglial Metabolism In Vitro. Metabolites.

[72] Tikhanovich I., Kuravi S., Artigues A., Villar M.T., Dorko K., Nawabi A., Roberts B., Weinman S.A. (2015). Dynamic Arginine Methylation of Tumor Necrosis Factor (TNF) Receptor-associated Factor 6 Regulates Toll-like Receptor Signaling. J. Biol. Chem..

[73] Medrano-Jimenez E., Jimenez-Ferrer Carrillo I., Pedraza-Escalona M., Ramirez-Serrano C.E., Alvarez-Arellano L., Cortes-Mendoza J., Herrera-Ruiz M., Jimenez-Ferrer E., Zamilpa A., Tortoriello J. (2019). Malva parviflora extract ameliorates the deleterious effects of a high fat diet on the cognitive deficit in a mouse model of Alzheimer’s disease by restoring microglial function via a PPAR-γ-dependent mechanism. J. Neuroinflamm..

[74] Liu Z., Zhao X., Liu B., Liu A.J., Li H., Mao X., Wu B., Bi K.S., Jia Y. (2014). Jujuboside A, a neuroprotective agent from semen Ziziphi Spinosae ameliorates behavioral disorders of the dementia mouse model induced by Aβ 1-42. Eur. J. Pharmacol..

[75] Yu T., Xie W., Sun Y. (2019). Oridonin inhibits LPS-induced inflammation in human gingival fibroblasts by activating PPARγ. Int. Immunopharmacol..

[76] Li-Hua D., Yan L., Shi-Ji W., Guang W., Lu-Lu S., Xue-Feng P., Pengda S. (2017). Esculentoside A inhibits LPS-induced BV2 microglia activation through activating PPAR-γ. Eur. J. Pharmacol..

[77] Huang C., Yang Y., Li W.X., Wu X.Q., Li X.F., Ma T.T., Zhang L., Meng X.M., Li J. (2015). Hyperin attenuates inflammation by activating PPAR-γ in mice with acute liver injury (ALI) and LPS-induced RAW264.7 cells. Int. Immunopharmacol..

[78] Lai J., Hu M., Wang H., Hu M., Long Y., Miao M.X., Li J.C., Wang X.B., Kong L.Y., Hong H. (2014). Montelukast targeting the cysteinyl leukotriene receptor 1 ameliorates Aβ1-42-induced memory impairment and neuroinflammatory and apoptotic responses in mice. Neuropharmacology.

[79] Attaluri S., Upadhya R., Kodali M., Madhu L.N., Upadhya D., Shuai B., Shetty A.K. (2022). Brain-Specific Increase in Leukotriene Signaling Accompanies Chronic Neuroinflammation and Cognitive Impairment in a Model of Gulf War Illness. Front. Immunol..

[80] Kanaoka Y., Austen K.F. (2019). Roles of cysteinyl leukotrienes and their receptors in immune cell-related functions. Adv. Immunol..

[81] Baraban S.C., Taylor M.R., Castro P.A., Baier H. (2005). Pentylenetetrazole induced changes in zebrafish behavior, neural activity and c-fos expression. Neuroscience.

[82] Novoa B., Bowman T.V., Zon L., Figueras A. (2009). LPS response and tolerance in the zebrafish (Danio rerio). Fish. Shellfish. Immunol..

[83] Widder M., Carbaugh C., van der Schalie W., Miller R., Brennan L., Moore A., Campbell R., Akers K., Ressner R., Martin M. (2024). Identification of Potential Sepsis Therapeutic Drugs Using a Zebrafish Rapid Screening Approach. Life.

[84] Yu L., Yan J., Sun Z. (2017). D-limonene exhibits anti-inflammatory and antioxidant properties in an ulcerative colitis rat model via regulation of iNOS, COX-2, PGE2 and ERK signaling pathways. Mol. Med. Rep..

[85] Cobourne-Duval M.K., Taka E., Mendonca P., Soliman K.F.A. (2018). Thymoquinone increases the expression of neuroprotective proteins while decreasing the expression of pro-inflammatory cytokines and the gene expression NFκB pathway signaling targets in LPS/IFNγ -activated BV-2 microglia cells. J. Neuroimmunol..

[86] Yang R., Liu S., Zhou J., Bu S., Zhang J. (2017). Andrographolide attenuates microglia-mediated Aβ neurotoxicity partially through inhibiting NF-κB and JNK MAPK signaling pathway. Immunopharmacol. Immunotoxicol..

[87] Roberto D., Micucci P., Sebastian T., Graciela F., Anesini C. (2010). Antioxidant activity of limonene on normal murine lymphocytes: Relation to H2O2 modulation and cell proliferation. Basic. Clin. Pharmacol. Toxicol..

[88] Porres-Martinez M., Gonzalez-Burgos E., Carretero M.E., Gomez-Serranillos M.P. (2015). Major selected monoterpenes α-pinene and 1,8-cineole found in Salvia lavandulifolia (Spanish sage) essential oil as regulators of cellular redox balance. Pharm. Biol..

[89] Tiwari M., Kakkar P. (2009). Plant derived antioxidants—Geraniol and camphene protect rat alveolar macrophages against t-BHP induced oxidative stress. Toxicol. Vitr..

[90] Staton Laws Iii J., Smid S.D. (2024). Sesquiterpene-evoked phytochemical toxicity in PC12 neuronal cells reveals a variable degree of oxidative stress and α-tocopherol and glutathione-dependent protection. Curr. Res. Toxicol..

[91] Huang Y., Xiang P., Chen Y., Pan Q., Yuan K. (2024). Alantolactone facilitates ferroptosis in non-small cell lung cancer through promoting FTH1 ubiquitination and degradation. Chem. Biol. Drug Des..

[92] Kovalevich J., Langford D. (2013). Considerations for the use of SH-SY5Y neuroblastoma cells in neurobiology. Methods Mol. Biol..

[93] Tremblay R.G., Sikorska M., Sandhu J.K., Lanthier P., Ribecco-Lutkiewicz M., Bani-Yaghoub M. (2010). Differentiation of mouse Neuro 2A cells into dopamine neurons. J. Neurosci. Methods.

[94] Bocchini V., Mazzolla R., Barluzzi R., Blasi E., Sick P., Kettenmann H. (1992). An immortalized cell line expresses properties of activated microglial cells. J. Neurosci. Res..

[95] Zhang M., Qian C., Zheng Z.G., Qian F., Wang Y., Thu P.M., Zhang X., Zhou Y., Tu L., Liu Q. (2018). Jujuboside A promotes Aβ clearance and ameliorates cognitive deficiency in Alzheimer’s disease through activating Axl/HSP90/PPARγ pathway. Theranostics.

[96] Piantadosi C.A., Withers C.M., Bartz R.R., MacGarvey N.C., Fu P., Sweeney T.E., Welty-Wolf K.E., Suliman H.B. (2011). Heme oxygenase-1 couples activation of mitochondrial biogenesis to anti-inflammatory cytokine expression. J. Biol. Chem..

[97] Choi S., Nguyen V.T., Tae N., Lee S., Ryoo S., Min B.S., Lee J.H. (2014). Anti-inflammatory and heme oxygenase-1 inducing activities of lanostane triterpenes isolated from mushroom Ganoderma lucidum in RAW264.7 cells. Toxicol. Appl. Pharmacol..

[98] Hwangbo C., Lee H.S., Park J., Choe J., Lee J.H. (2009). The anti-inflammatory effect of tussilagone, from Tussilago farfara, is mediated by the induction of heme oxygenase-1 in murine macrophages. Int. Immunopharmacol..

[99] Lee W.Y., Chen Y.C., Shih C.M., Lin C.M., Cheng C.H., Chen K.C., Lin C.W. (2014). The induction of heme oxygenase-1 suppresses heat shock protein 90 and the proliferation of human breast cancer cells through its byproduct carbon monoxide. Toxicol. Appl. Pharmacol..

[100] Na J.Y., Song K., Lee J.W., Kim S., Kwon J. (2016). 6-Shogaol has anti-amyloidogenic activity and ameliorates Alzheimer’s disease via CysLT1R-mediated inhibition of cathepsin B. Biochem. Biophys. Res. Commun..

[101] Wang X., Gan W., Kang M., Lv C., Zhao Z., Wu Y., Zhang X., Wang R. (2024). Asthma aggravates alzheimer’s disease by up-regulating NF-κB signaling pathway through LTD4. Brain Res..

[102] Sauer A., Hartung T. (1994). Ltd_4_ Augments Tnf Release in-Vivo and in-Vitro. Agents Actions.

[103] Thompson C., Cloutier A., Bosse Y., Thivierge M., Gouill C.L., Larivee P., McDonald P.P., Stankova J., Rola-Pleszczynski M. (2006). CysLT1 receptor engagement induces activator protein-1- and NF-κB-dependent IL-8 expression. Am. J. Respir. Cell Mol. Biol..

[104] Mulyaningsih S., Youns M., El-Readi M.Z., Ashour M.L., Nibret E., Sporer F., Herrmann F., Reichling J., Wink M. (2010). Biological activity of the essential oil of Kadsura longipedunculata (Schisandraceae) and its major components. J. Pharm. Pharmacol..

[105] Mascayano C., Munoz-Osses M., Navarrete E., Torres P., Torres-Gonzalez S., Morales P., Huidobro-Toro J.P. (2024). Natural pentacyclic triterpenoid as allosteric modulators of human 5-lipoxygenase with potential anti-inflammatory activity. J. Biomol. Struct. Dyn..

[106] El-Dakhakhny M., Madi N.J., Lembert N., Ammon H.P. (2002). Nigella sativa oil, nigellone and derived thymoquinone inhibit synthesis of 5-lipoxygenase products in polymorphonuclear leukocytes from rats. J. Ethnopharmacol..

[107] Chen L., Yang Y., Li C.T., Zhang S.R., Zheng W., Wei E.Q., Zhang L.H. (2015). CysLT2 receptor mediates lipopolysaccharide-induced microglial inflammation and consequent neurotoxicity in vitro. Brain Res..

[108] Kyritsis N., Kizil C., Zocher S., Kroehne V., Kaslin J., Freudenreich D., Iltzsche A., Brand M. (2012). Acute inflammation initiates the regenerative response in the adult zebrafish brain. Science.

[109] Butler C.T., Reynolds A.L., Tosetto M., Dillon E.T., Guiry P.J., Cagney G., O’Sullivan J., Kennedy B.N. (2017). A Quininib Analogue and Cysteinyl Leukotriene Receptor Antagonist Inhibits Vascular Endothelial Growth Factor (VEGF)-independent Angiogenesis and Exerts an Additive Antiangiogenic Response with Bevacizumab. J. Biol. Chem..

[110] Slater K., Bosch R., Smith K.F., Jahangir C.A., Garcia-Mulero S., Rahman A., O’Connell F., Piulats J.M., O’Neill V., Horgan N. (2022). 1,4-dihydroxy quininib modulates the secretome of uveal melanoma tumour explants and a marker of oxidative phosphorylation in a metastatic xenograft model. Front Med..

[111] Tesfaye B.A., Hailu H.G., Zewdie K.A., Ayza M.A., Berhe D.F. (2021). Montelukast: The New Therapeutic Option for the Treatment of Epilepsy. J. Exp. Pharmacol..

[112] Lenz Q.F., Arroyo D.S., Temp F.R., Poersch A.B., Masson C.J., Jesse A.C., Marafiga J.R., Reschke C.R., Iribarren P., Mello C.F. (2014). Cysteinyl leukotriene receptor (CysLT) antagonists decrease pentylenetetrazol-induced seizures and blood-brain barrier dysfunction. Neuroscience.

[113] Fleck J., Temp F.R., Marafiga J.R., Jesse A.C., Milanesi L.H., Rambo L.M., Mello C.F. (2016). Montelukast reduces seizures in pentylenetetrazol-kindled mice. Braz. J. Med. Biol. Res..

[114] Shaw P.A.G., Panda S.K., Stanca A., Luyten W. (2022). Optimization of a locomotion-based zebrafish seizure model. J. Neurosci. Methods.

[115] Zhang B., Wang L., Ji X., Zhang S., Sik A., Liu K., Jin M. (2020). Anti-Inflammation Associated Protective Mechanism of Berberine and its Derivatives on Attenuating Pentylenetetrazole-Induced Seizures in Zebrafish. J. Neuroimmune Pharmacol..

[116] Sturgeon M.L., Langton R., Sharma S., Cornell R.A., Glykys J., Bassuk A.G. (2021). The opioid antagonist naltrexone decreases seizure-like activity in genetic and chemically induced epilepsy models. Epilepsia Open.

[117] Murugan R., Ramya Ranjan Nayak S.P., Haridevamuthu B., Priya D., Chitra V., Almutairi B.O., Arokiyaraj S., Saravanan M., Kathiravan M.K., Arockiaraj J. (2024). Neuroprotective potential of pyrazole benzenesulfonamide derivative T1 in targeted intervention against PTZ-induced epilepsy-like condition in in vivo zebrafish model. Int. Immunopharmacol..

[118] Oosterhof N., Boddeke E., van Ham T.J. (2015). Immune cell dynamics in the CNS: Learning from the zebrafish. Glia.

[119] Ji S.Y., Cha H.J., Molagoda I.M.N., Kim M.Y., Kim S.Y., Hwangbo H., Lee H., Kim G.Y., Kim D.H., Hyun J.W. (2021). Suppression of Lipopolysaccharide-Induced Inflammatory and Oxidative Response by 5-Aminolevulinic Acid in RAW 264.7 Macrophages and Zebrafish Larvae. Biomol Ther.

[120] Kanwal Z., Wiegertjes G.F., Veneman W.J., Meijer A.H., Spaink H.P. (2014). Comparative studies of Toll-like receptor signalling using zebrafish. Dev. Comp. Immunol..

[121] Loes A.N., Hinman M.N., Farnsworth D.R., Miller A.C., Guillemin K., Harms M.J. (2021). Identification and Characterization of Zebrafish Tlr4 Coreceptor Md-2. J. Immunol..

[122] Bartikova H., Hanusova V., Skalova L., Ambroz M., Bousova I. (2014). Antioxidant, pro-oxidant and other biological activities of sesquiterpenes. Curr. Top. Med. Chem..

[123] Jones A.C., Pinki F., Stewart G.S., Costello D.A. (2021). Inhibition of Urea Transporter (UT)-B Modulates LPS-Induced Inflammatory Responses in BV2 Microglia and N2a Neuroblastoma Cells. Neurochem. Res..

